# The responses of soil organic carbon and total nitrogen to chemical nitrogen fertilizers reduction base on a meta-analysis

**DOI:** 10.1038/s41598-022-18684-w

**Published:** 2022-09-29

**Authors:** Chuanzong Li, Oluwaseun Olayemi Aluko, Guang Yuan, Jiayi Li, Haobao Liu

**Affiliations:** Tobacco Research Institute, Chinese Academy of Agricultural Sciences (CAAS), Qingdao, China

**Keywords:** Ecology, Environmental sciences

## Abstract

Soil organic carbon (SOC), total nitrogen (TN), and their ratio (C:N) play important roles in preserving soil fertility, and their values are closely related to fertilizer use. However, the overall trend and magnitude of changes in SOC, TN and C:N in response to chemical nitrogen fertilizers reduction remain inconclusive. Here, the meta-analysis conducted comparisons at 48 sites covering various cropping system, soil type, and climatic regions of China to investigate the responses of SOC, TN and C:N to chemical nitrogen fertilizers reduction. The results showed that chemical nitrogen fertilizers reduction decreased SOC by 2.76 ± 0.3% and TN by 4.19 ± 0.8%, and increased the C:N by 6.11 ± 0.9% across all the database. Specifically, the reduction of chemical nitrogen without adding organic nitrogen fertilizers would reduce SOC and TN by 3.83% and 11.46% respectively, while they increased SOC and TN by 4.92% and 8.33% respectively with organic fertilizers supplement, suggesting that organic fertilizers could cover the loss of SOC, TN induced by chemical nitrogen fertilizers reduction. Medium magnitude (20–30%) of chemical nitrogen fertilizers reduction enhanced SOC by 6.9%, while high magnitude (≧30%) and total (100%) of chemical nitrogen fertilizers reduction significantly decreased SOC by 3.10% and 7.26% respectively. Moreover, SOC showed a negative response to nitrogen fertilizers reduction at short-term duration (1–2 years), while the results converted under medium-long-termThis system analysis fills the gap on the effects of fertilizer reduction on soil organic carbon and nitrogen at the national scale, and provides technical foundation for the action of reducing fertilizer application while increase efficiency.

## Introduction

Fertilization is considered as one of the most significant agronomic practices for increasing crop yields and food security, especially for the application of chemical nitrogen fertilizers^1,2^. Large quantities of chemical nitrogen fertilizers have been employed to increase the land productivity^3,4^. Until 2013, the global composition of nitrogen fertilizers has reached up to 107.6 Tg N year^−1^, but cannot be fully absorbed by crops^5^. And the successive and considerable application of chemical nitrogen fertilizers has led to serious environment problems including soil degradation, soil eutrophication, and greenhouse effect^6,7^. Therefore, chemical nitrogen fertilizers reduction has aroused the concern of the world for the sustainable development of agriculture^8–10^.

The effects of chemical nitrogen fertilizers reduction on SOC, TN and C:N has been evaluated in specific regions. For example, Cheng et al.^10^ described that SOC in a fluvo-aquic brown soil had a significantly decrease under the treatment of 20% chemical nitrogen fertilizers reduction after one year with maize. However, the study of Ning et al.^11^ stated that there was no significant difference about SOC after 20% chemical nitrogen fertilizers reduction in a 10-season continually vegetable planted field. As for TN, researches have reported variations under different nitrogen application regime^12^. No significant difference about TN was observed with 25% reduction of chemical nitrogen fertilizers in the study of Liu et al.^13^. However, the results of regional-specific differences did not clarify the overall impact on national carbon and nitrogen after chemical fertilizer reduction. The overall trend and magnitude of changes in SOC, TN and C:N in response to chemical nitrogen fertilizers reduction are still unclear. Taking into account differences in climatic conditions, soil types, farming systems and trial times, national data are needed to assess the response of fertilizer reduction to SOC, TN and C:N.

As a powerful statistical method for comparing and integrating results from multiple studies, meta-analysis which could overcome the limitation of the highly variability of different studies has been widely applied for the comprehensive analysis of randomized controlled trials about clinical researches^14,15^. Currently, meta-analysis has made a revolutionary effect in the field of soil science, and great achievements have been acquired^16,17^. Du et al.^18^ employed a meta-analysis to assess the effects of no-till on the SOC storage compared to conventional tillage, and found that the influences of no-till on SOC were overemphasized in China. The responses of microbial biomass carbon and nitrogen have been estimated to experimental warming in the study of Xu and Yuan^19^, and they demonstrated that experimental warming significantly increased microbial biomass. Each of these meta-analyses only focused on farming system or analyzed in limited region. Therefore, a nationwide meta-analysis with SOC, TN and C:N response to chemical nitrogen fertilizers reduction is necessary.

In this study, the meta-analysis was established to assess the relative changes in SOC, TN and C:N based on 295 comparisons between chemical nitrogen fertilizers reduction treatments and conventional treatments from 48 sites covering various cropping systems, soil types, and climatic regions of China. The objectives of this study were to (i) explore the overall trend and magnitude of changes in SOC, TN and C:N in response to chemical nitrogen fertilizers reduction; (ii) clarify the response of SOC, TN and C:N to different subcategories including: reduction pattern, experimental duration, reduction magnitude, soil use; and (iii) identify the relationships between environmental factors and the response of SOC, TN and C:N.

## Results and discussion

### The overall magnitude of changes in SOC, TN, and C:N in response to chemical nitrogen fertilizers reduction

**Figure 1:**
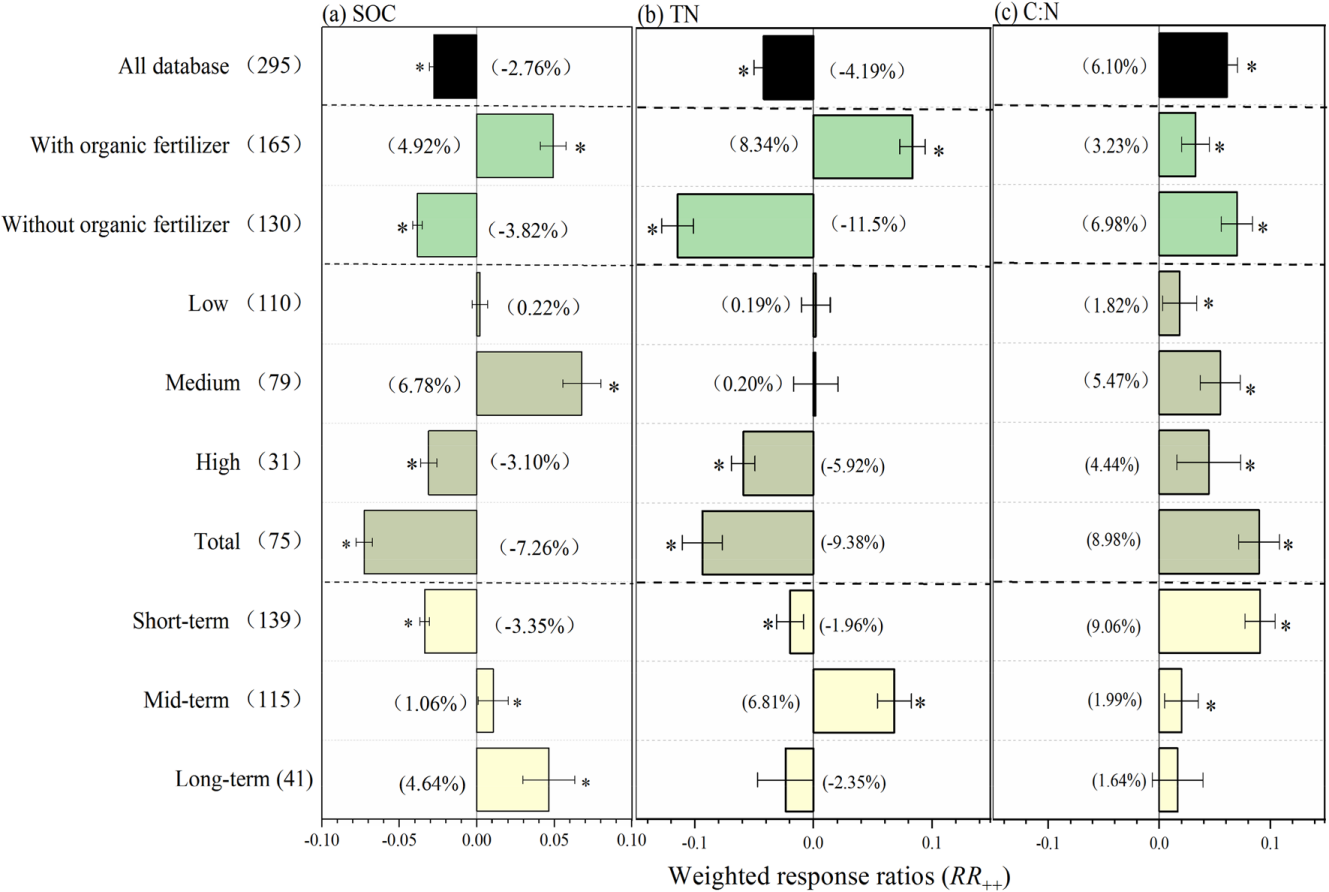
The weighted response ratio (*RR*_++_) for the responses to chemical nitrogen fertilizers of soil organic carbon (SOC, **a**), total nitrogen (TN, **b**), and their ratios (C:N, **c**). Bars denote the overall mean response ratio *RR*_++_ and 95% confidence intervals (CI). The star (*) indicates significance when the 95% CI that do not go across the zero line. The vertical lines are drawn at ln*RR* = 0. The value represents independent sample size.

The results showed that chemical nitrogen fertilizers reduction significantly decreased SOC and TN by 2.76% and 4.19% respectively, while increased C:N by 6.11% across all database (Fig. 1). SOC mainly derives from crop residues and secretions which closely related to crops growths, and crops growths were affected by fertilization, especially nitrogen fertilization^20,21^. The reduction of chemical nitrogen fertilizer led to poor crop growth, which reduced the amount of crop residues return, and then decreased SOC. Similarly, TN from crops was reduced due to poor crop growth. In addition, the reduction of chemical nitrogen fertilizers directly reduced the input of soil nitrogen. The increase of C:N was the result of the decrease of TN being greater than that of SOC. The responses of C:N to chemical nitrogen fertilizers reduction enhanced the comprehension of the couple relationship between SOC and TN, which was beneficial to the evolution of the C-N coupling models. Moreover, the accuracy of C-N coupling models depends on the precise quantification of the responses of SOC and TN to nitrogen fertilization. And our results accurately quantified the difference responses of SOC and TN to different nitrogen fertilization regimes, thus optimizing the C-N coupling models.

### Responses of SOC, TN and C:N to chemical nitrogen fertilizers reduction magnitude

When grouped by chemical nitrogen fertilizers reduction magnitude, SOC showed a significant increase by 6.9% in medium magnitude, while SOC was significantly decreased by 3.10% and 7.26% in high and total magnitude respectively (Fig. 1a). Liu and Greaver^22^ also stated the reduction of medium nitrogen fertilizer increased the average microbial biomass from 15 to 20%, thereby increasing the SOC content. Previous studies had reported that there were strong positive correlations between soil organic matter and soil microbial biomass in both the agricultural ecosystem and natural ecosystem^23,24^. Numerous researchers have demonstrated the significance of nitrogen availability in soil for the plant biomass across most ecosystems^25,26^. Moreover, nitrogen deficient would inhibit the activity of extracellular enzymes and root activities^27^. Generally, soil degradation caused by continuous rising chemical nitrogen fertilizers application may inhibit the growth of crops and ultimately reduce the SOC^28^.

TN showed no significant difference in low and medium chemical nitrogen fertilizers reduction magnitude (*p* > 0.05), while TN in high magnitude and total chemical nitrogen fertilizers reduction magnitude exhibited a decrease with 3.10% and 9.37% respectively (Fig. 1b). Numerous studies described that the amount of nitrogen fertilizers used in China was higher than the demand of N for crop, which caused serious N leaching and runoff^29,30^. Chemical nitrogen fertilizers in low and medium magnitude would not decrease the TN of soil by reducing N leaching and runoff. However, the residual nitrogen in soil cannot meet the requirement for the sustainable growth of plant with litter or without exogenous nitrogen supplement, which resulted in the decrease of TN in high and total chemical nitrogen fertilizers magnitude. Consequently, optimal nitrogen fertilizers application rates will take into account crops yield and environment friendliness.

Additionally, C:N had a significant increase with ranging from 1.82% to 8.98% under the four chemical nitrogen fertilizers reduction magnitude (Fig. 1c), suggesting the relative increase of SOC compared to TN. Previous studies have revealed that C:N had significantly influence on the soil bacterial community structures^31^. And there were also considerable studies indicated that chemical nitrogen fertilizers have impact on the soil bacterial communities^32,33^. We may speculate that the change of C:N would bring about the variations of soil bacteria communities under the chemical nitrogen fertilizers regimes.

### Responses of SOC, TN, and C:N to chemical nitrogen fertilizers reduction duration

Negative response of SOC to short-term chemical nitrogen fertilizers reduction was observed in our study, which was consistent with the study of Gong, et al.^34^ that chemical nitrogen fertilizers reduction decreased SOC by reducing crop-derived carbon by one year. However, SOC was significantly increased by 1.06% and 4.65% at mid-term and long-term chemical nitrogen fertilizers reduction respectively, which was similar with the findings of Ning, et al.^11^ that SOC was significantly increased under more than 5 years of chemical nitrogen fertilizers reduction treatment. TN was significantly decreased by 1.96% at short-term chemical nitrogen fertilizers reduction duration, while the results converted at mid-term chemical nitrogen fertilizers reduction duration. The effect of long-term chemical nitrogen fertilizers reduction on TN was not significant (*p* > 0.05). The divergent response of TN to different chemical nitrogen fertilizers duration was mainly caused by the various treatments. In terms of C:N, a greater positive response was observed at short-term chemical nitrogen fertilizers duration (9.06%) than mid-term and long-term duration (1.99%). Moreover, with the prolongation of the chemical reduction time of nitrogen, the response ratio tends to zero, suggesting that the effect of chemical fertilizers gradually decrease. This may be ascribed to the buffer capacity of soil to resist the changes from external environment, including nutrients, pollutants, and redox substances^35^.

### Responses of SOC, TN, and C:N to different chemical nitrogen fertilizers reduction patterns

**Figure 2:**
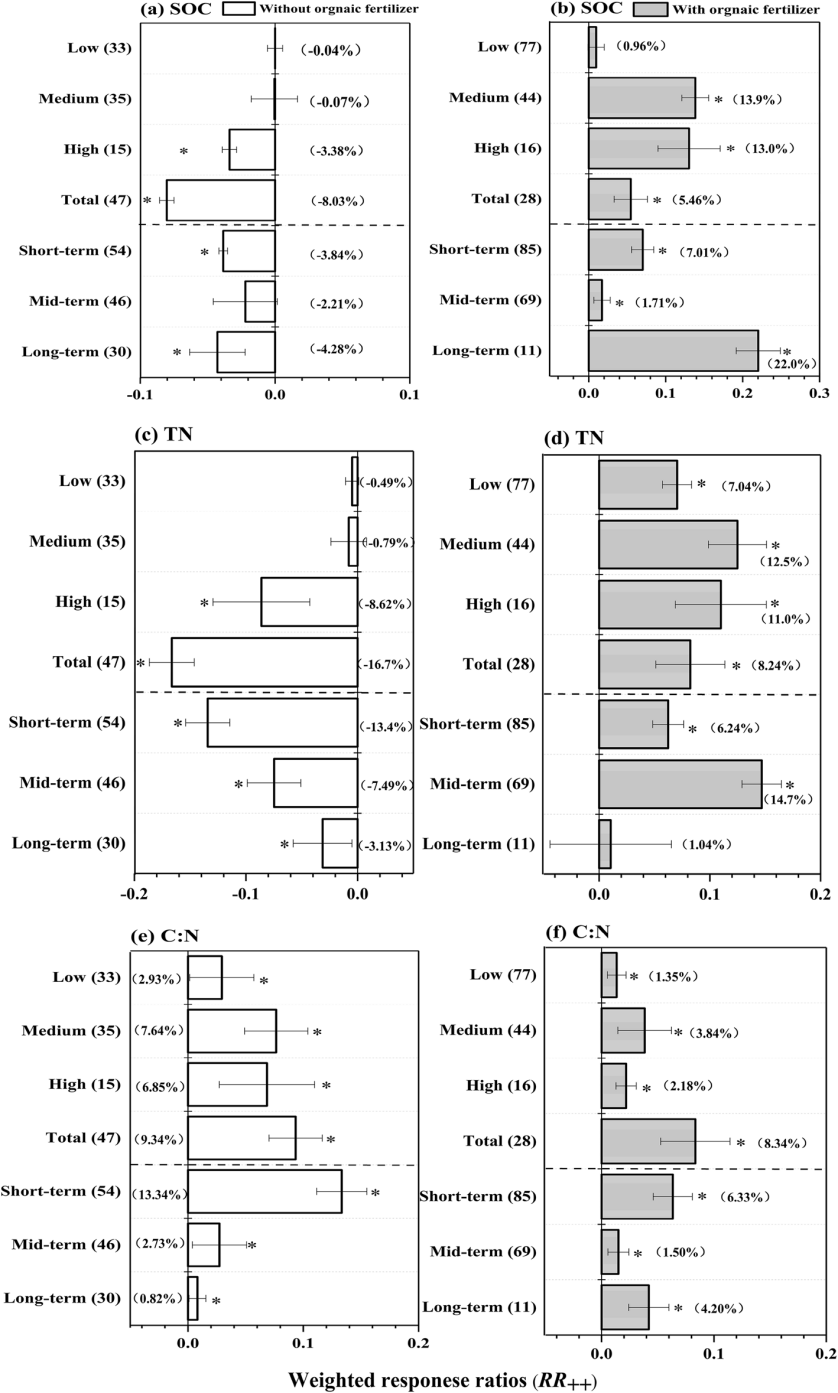
The weighted response ratio (*RR*_++_) for the responses to chemical nitrogen fertilizers of soil organic carbon (SOC, **a**), total nitrogen (TN, **b**), and their ratios (C:N, **c**) under the two patterns (with organic fertilizers ; without organic fertilizers). Bars denote the overall mean response ratio *RR*_++_ and 95% confidence intervals (CI). The star (*) indicates significance when the 95% CI that do not go across the zero line. The vertical lines are drawn at ln*RR* = 0. The values represent independent sample size.

Under the pattern of chemical nitrogen fertilizers reduction without organic fertilizers supplement, SOC and TN significantly decreased by 3.83% and 11.46% respectively, however, chemical nitrogen fertilizers reduction with organic fertilizers supplement significantly increased SOC and TN by 4.92% and 8.33% respectively. Moreover, C:N significantly increased under the two chemical nitrogen fertilizers patterns (*p* < 0.05) (Fig. 1). To further analysis the importance of organic fertilizers application on SOC, TN and C:N, the total database was divided into two categories including reduction duration and reduction magnitude of each pattern. And the responses SOC, TN and C:N under the two chemical nitrogen fertilizers patterns of each categories were shown in the Fig. 2. In the pattern of chemical nitrogen fertilizers without organic fertilizers supplement, low and medium magnitude of chemical nitrogen fertilizers had no significantly influence on the response of SOC (*p* > 0.05), but there was a negative effect on SOC in high and total magnitude (*p* < 0.05). In terms of chemical fertilizer reduction duration, chemical nitrogen fertilizers reduction decreased SOC by 3.8% and 4.2% at short and long term chemical nitrogen fertilizers duration respectively, while SOC showed no significantly decrease at mid-term duration (*p* > 0.05). The no significant decrease at mid-term duration might result from the limited information reported in original studies of this meta-analysis^36^. TN showed no significant response to chemical nitrogen fertilizers without organic fertilizers supplement in the low and medium magnitude (*p* > 0.05). However, TN was significantly decreased by 8.62% and 16.7% respectively in the high and total magnitude. When regarding to chemical nitrogen fertilizers reduction duration, TN was significantly reduced at all of the categories, ranging from 3.13% to 13.4% (Fig. 2c). In the pattern of chemical nitrogen fertilizers reduction with organic fertilizers supplement, chemical nitrogen fertilizers reduction at medium, high, and total magnitudes significantly increased SOC by 13.85%, 13.03%, and 5.46%respectively, however, the response of SOC in the low chemical nitrogen fertilizers magnitude was not significant. Chemical nitrogen fertilizers reduction duration significantly increased SOC by 7.01%, 1.71%, and 22.02% in the short-term, mid-term, and long-term respectively. Comparatively, TN showed a significantly increase in most chemical nitrogen fertilizers categories expect for the long-term chemical nitrogen fertilizers duration, with an increasing from 4.90% to 14.69% (Fig. 2d).

Organic fertilizers were mainly derived from animal manure or crops straws, which contained large amount of organic matter and nitrogen elements^37,38^. The application of organic fertilizers increased the input of SOC and TN directly. Moreover, organic fertilizer could promote the growth of crops by releasing phenols, vitamins, enzymes, auxins and other substances during the decomposition process, thus the SOC derived from crops would be increased^37,39^. In addition, organic fertilizers provide various nutrients for microbial reproduction, which increase the microbial population and organic carbon and total nitrogen content^37^. More importantly, the application of organic fertilizers could improve organic carbon sequestration and maintain its stability in aggregates, thereby reducing losses of SOC and TN^40^.

C:N showed an increase under all of the chemical nitrogen fertilizers reduction with organic fertilizer supplement. The positive response of C:N to organic fertilizer supplement may be related to the higher C:N of organic fertilizer than soil. The average values of C:N of the commonly used organic fertilizers including animal manure, crop straws and biochar were 14, 60 and 30 respectively, while the C:N of soil was lower than 10 in average according to extensive literature researches^41^. Therefore, organic fertilizers would be a favorable alternative of chemical fertilizers for the sustainable development of agriculture.

### The correlation between the response of SOC, TN, and C:N and environmental variables

**Figure 3:**
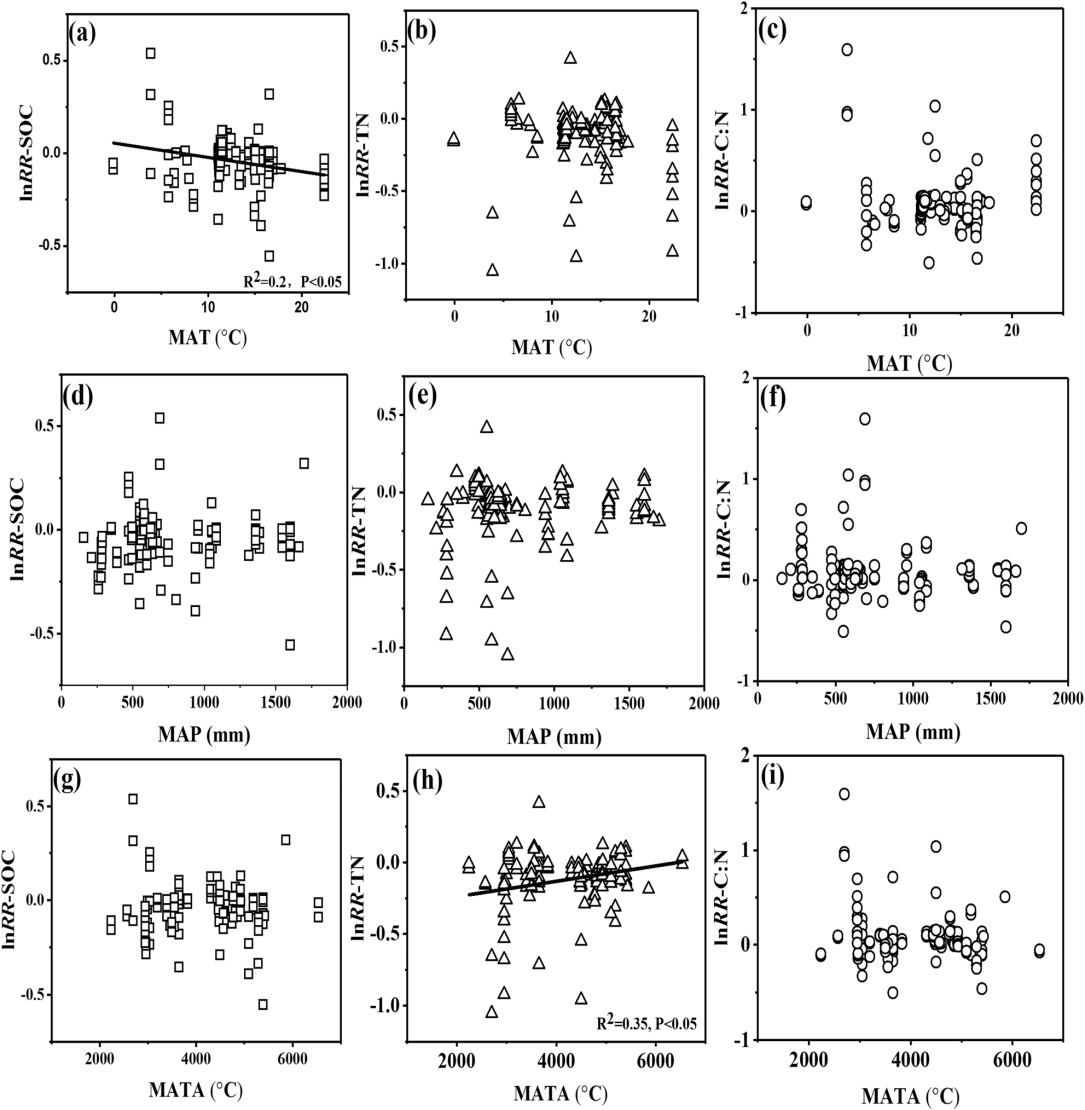
Relationship between mean annual temperature (MAT), mean annual precipitation (MAP), accumulated temperature above 10℃ (MATA) and the natural logarithm of the response ratio (lnRR) of soil organic carbon (SOC), total nitrogen (TN), and the ratio of SOC to TN (C:N). The black solid line shows the relationship between the ln*RR* of SOC, TN, C:N and environmental variables of all database of chemical nitrogen fertilizers without organic fertilizers supplement.

**Figure 4:**
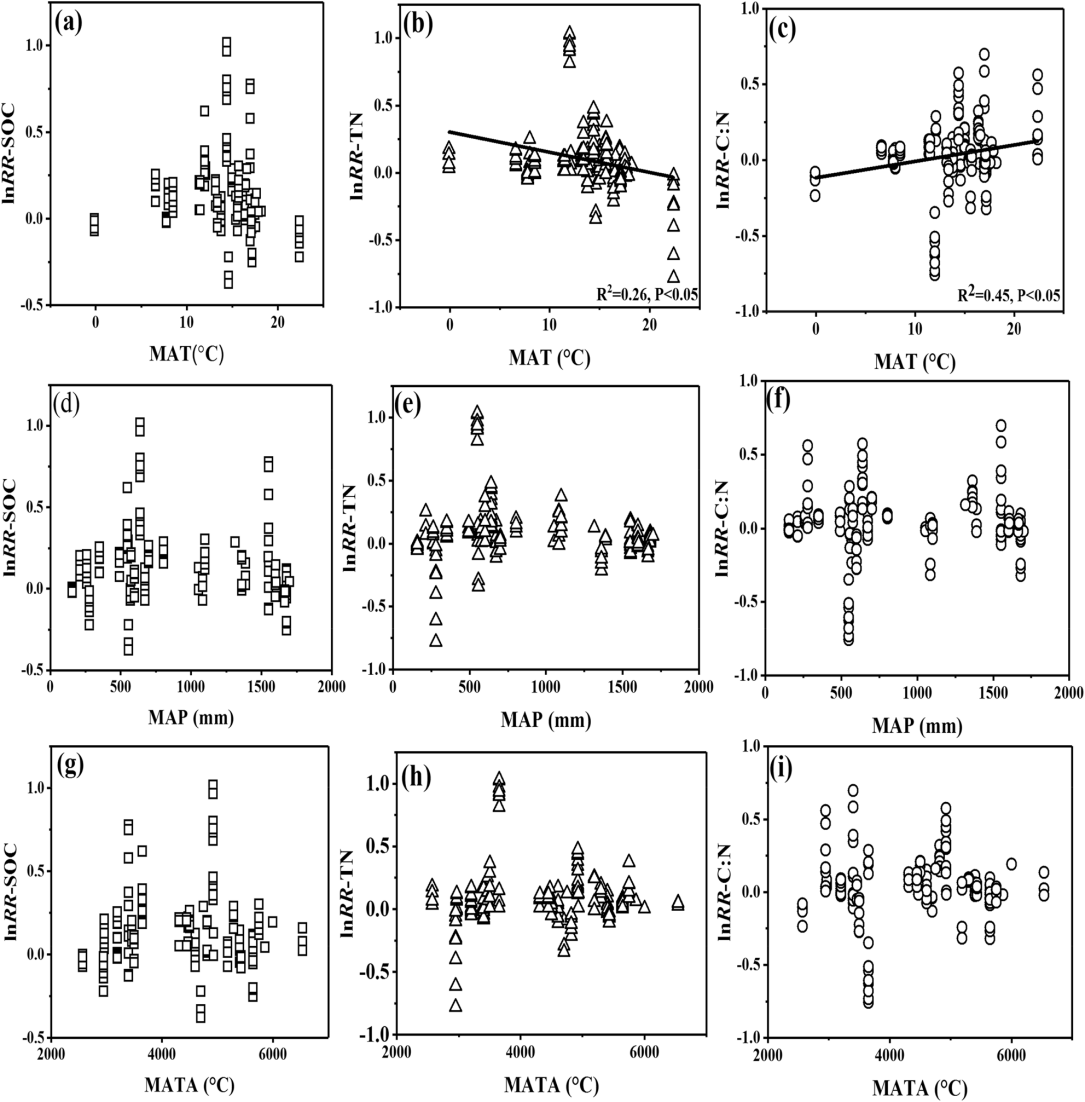
Relationship between mean annual temperature (MAT), mean annual precipitation (MAP), accumulated temperature above 10 °C (MATA) and the natural logarithm of the response ratio (lnRR) of soil organic carbon (SOC), total nitrogen (TN), and the ratio of SOC to TN (C:N). The black solid line shows the relationship between the lnRR of SOC, TN, C:N and environmental variables of all database of chemical nitrogen fertilizers with organic fertilizers supplement.

The analysis of linear regression was conducted to analyze the environmental variables including mean annual temperature (MAT), mean annual precipitation (MAP), accumulated temperature above 10 °C (MATA), which may exert influence on SOC, TN and C:N. No significant correlation among the ln*RR* of SOC, TN, C:N and environmental variables were observed among the whole database (*p* > 0.05; Fig. S1). Rule out the interference of organic fertilizers supplement, we analyzed the relationship between ln*RR* of SOC, TN, C:N and environmental variables as the Figures showed in Figs. 3 and 4 respectively. Under chemical nitrogen fertilizers without organic fertilizers supplement, there was a significant negative correlation between ln*RR* of SOC and MAT (*p* < 0.05, Fig. 3a) and a positively correlation between ln*RR* of TN and MATA (*p* < 0.05, Fig. 3h). However, there no significant relationship between ln*RR* of C:N and MAT, MAP, and MATA (*p* > 0.05). Apart from the significant negative correlation between the ln*RR* of SOC and MAT (*p* < 0.05, Fig. 4b) and the significant positive correlation between ln*RR* of C:N and MAT (*p* < 0.05, Fig. 4c), no other significant correlations was found between the ln*RR* of SOC, TN, C:N and environmental variables under the pattern of chemical nitrogen fertilizers with organic fertilizers supplement (*p* > 0.05, Fig. 4). The negative relationship between MAT and the effects of chemical nitrogen fertilizers reduction was mainly attributed to the high decomposition rate of soil organic matter under the conditions of high temperature^42,43^. MATA is a necessary requirement for the growth of crops, and MATA could accurately reflect the growth status of crops^44,45^. Although correlation did not prove causation, these findings suggested MATA had a significant effect on crop nitrogen.

## Conclusion

Our meta-analysis indicated that both SOC and TN exhibited negative responses to chemical nitrogen fertilizers reduction. Under low and medium magnitude of chemical nitrogen fertilizers reduction, SOC and TN showed different responses, however, SOC and TN showed significantly negative response to high and total magnitude. SOC and TN showed negative response to short-term chemical nitrogen fertilizers reduction duration, while the results converted under mid-term. It is worth noting that organic fertilizer application increase SOC and TN. Except for long-term chemical nitrogen reduction, C:N showed positive responses to all of the categories. These findings indicated that SOC and TN response to chemical nitrogen reduction were not constant and influenced by reduction magnitude, duration and patterns. Further studies are still needed to confirm the effects of fertilization on soil physical and chemical properties relying on more field experimental cases.

## Materials and methods

### Data collection

We reviewed journal articles published during 2000 to 2019, which concentrated on the responses of soil SOC and TN to chemical nitrogen fertilizers in China by searching several databases including Science Direct, Web of Science, Springer Link, and China Knowledge Resource Integrated Database, etc.. The search terms were incorporated with “nitrogen fertilizers”, “nitrogen reduction”, “nitrogen rate”, “and soil organic carbon”, “soil organic matter”, “total nitrogen”. The data was selected by a procedure of information retrieval, as follows: (i) the researches were conducted with side-by-side comparisons of control and treatment groups; (ii) the measurements were implemented under field conditions with at least a full year; (iii) the means, standard deviations (SD) and sample sizes of SOC, TN and C:N were reported or could be calculated. If SOM was reported, the SOC was calculated by the equation (SOC = SOM × 0.58); If standard error (SE) was provided in the paper, SD were calculated as:

1 $$ {\text{SD}} = {\text{SE}} \times \sqrt n $$

where *n* was the replicate number.

We assigned that SD was 1/10 of the means in cases there were no SE or SD reported^46^. (iv) chemical nitrogen fertilizers variables (reduction patterns, reduction magnitude, reduction duration, and soil use) must be described detailed in the researches. If the reported results included one more soil layers, the uppermost layer was only selected in the present research.

For each selected paper, we recorded the means of SOC, TN, and C:N of control and treatment groups respectively. In the case of displaying data graphically, GetData Graph Digitizer version 2.24 was applied to digitize the data. Moreover, the characteristics of the study sites including location, soil texture, experimental duration, annual precipitation, annual mean temperature, accumulated temperature above 10 °C, and crops types were also extracted.

**Figure 5:**
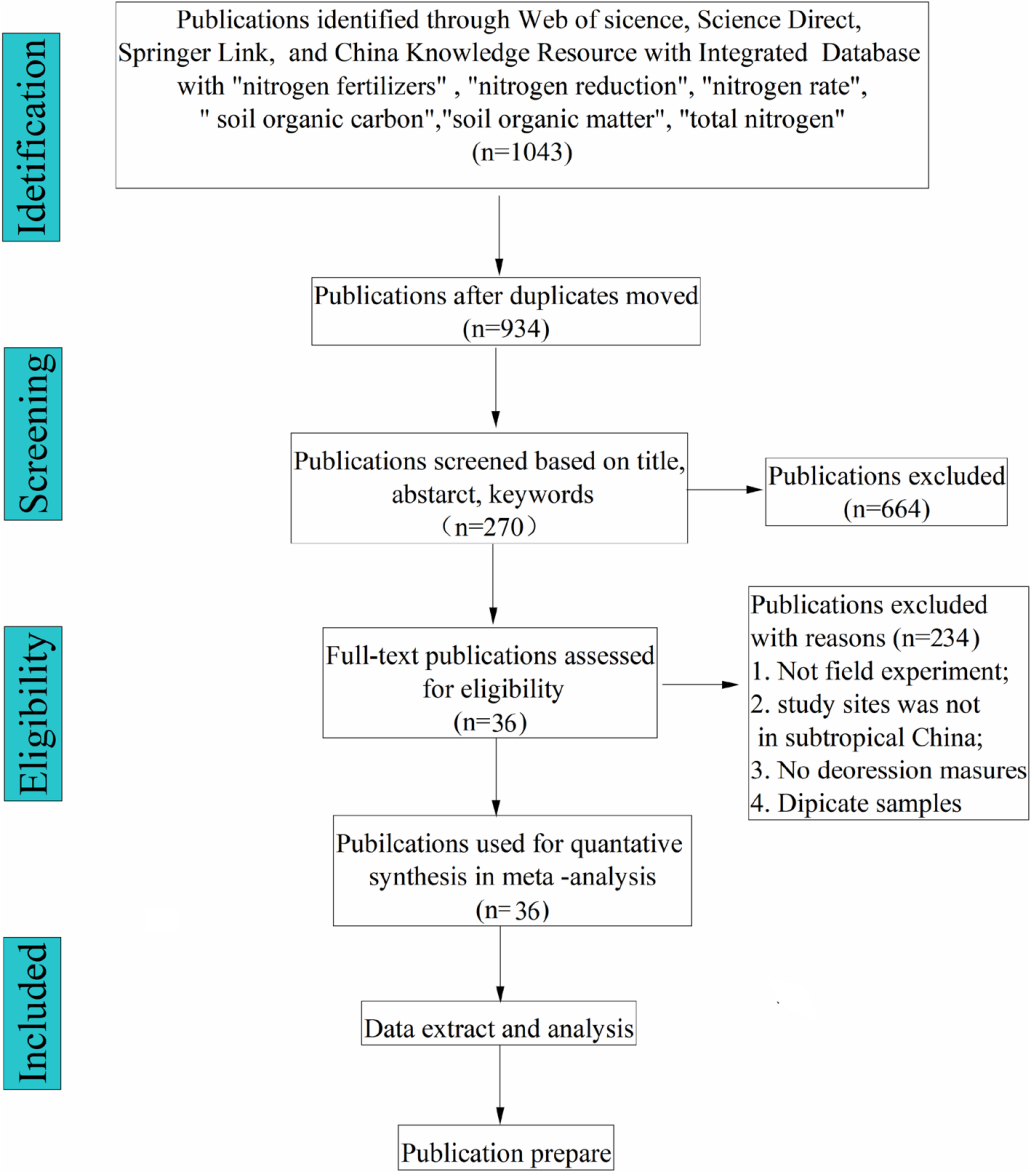
Flow chart of the present study.

**Figure 6:**
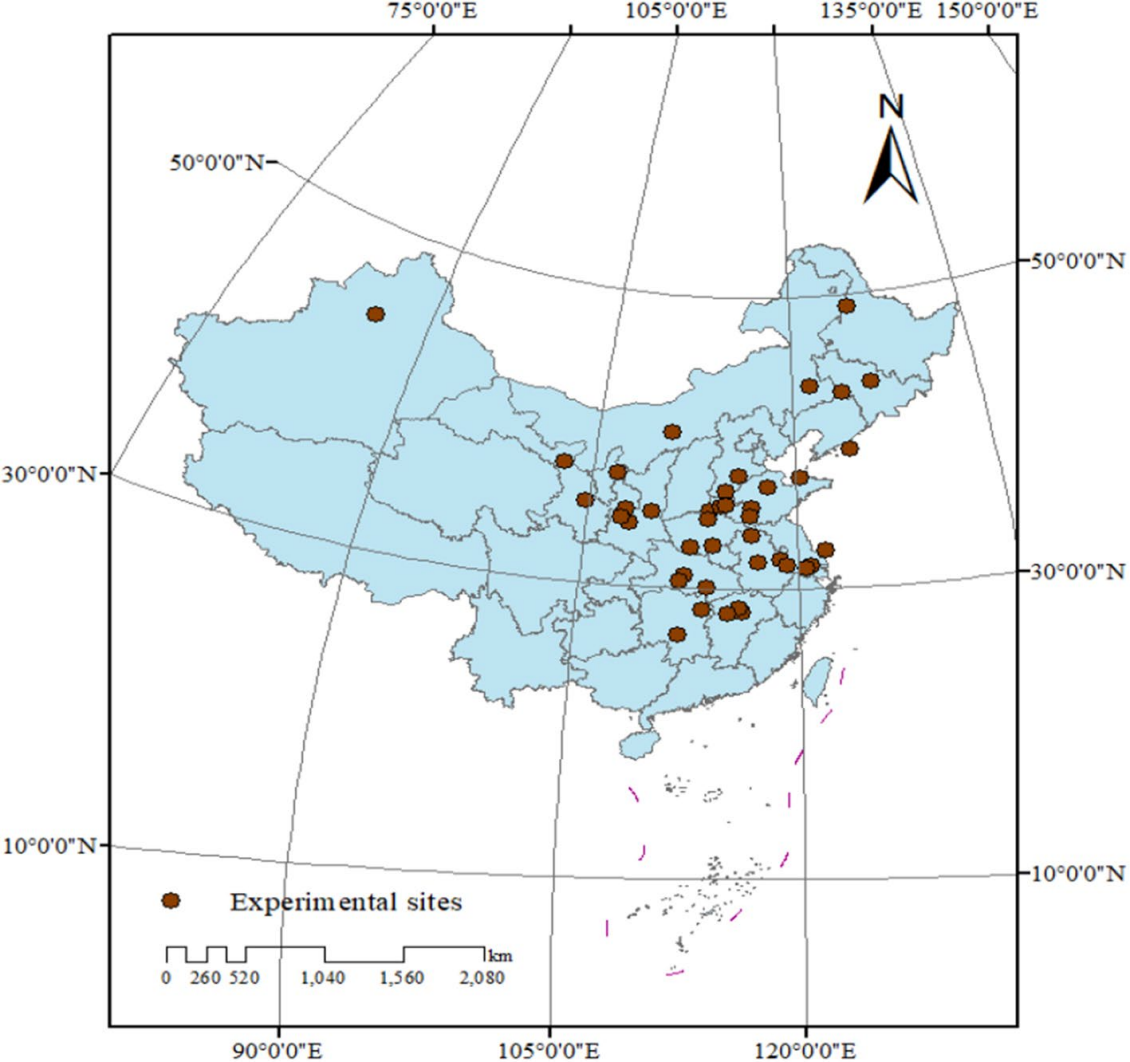
Spatial distribution map of chemical nitrogen fertilizers field experiment sites in China.

After filtering procedure (Fig. 5), 36 published papers consisted of peer-reviewed papers and dissertations from 48 sites in China were selected in the present study. The distribution of the experimental sites ranging from 85.6°E to 126.4°E, and 26.7°N to 49.2°N was presented in Fig. 6 and the detailed information was listed in the supplemental materials (Table S1). The database covered large ranges of mean annual temperature (from − 0.1 °C to 22.4 °C), mean annual precipitation (from 158 to 1700 mm), and accumulated temperature above 10 °C (from 2239 °C to 6539 °C) (Table S1). The crops planted in the experimental sites include corn, wheat, rice and cotton. The experimental duration ranged from 1 to 37 years, including 147 paired comparisons within 1–2 years, 94 comparisons within 3–4 years, and 41 comparisons more than 5 years.

To further explore the variations of SOC and TN induced by nitrogen chemical fertilizers reduction, all of the experimental data were partitioned into different subcategories (i) soil texture (sandy, loam, clay); (ii) experimental duration ( short-term experimental duration (1–2 years), mid-term experimental duration (3–4 years), and long-term experimental duration (≥ 5 years); (iii) reduction magnitude low reduction magnitude (≦20%), medium reduction magnitude (20%-30%), high reduction magnitude (≧30%), and total nitrogen chemical fertilizers reduction or no chemical N fertilizer application (100%); (iv) reduction pattern (without organic fertilizers supplement, with organic fertilizers supplement).

### Data analysis

The response ratio (*RR*) is an index used to evaluate the effects of experimental to each variable^47,48^. For a given variable, *RR* is determined as the ratio of the mean value the treatment group (*Mt*) to that under the control group (*Mc*). The calculation formula of *RR* was as follow:

2 $$ RR = M_{t} /M_{c} $$

where Mt and Mc represent the mean values of the treatments and control groups, respectively.

The ln*RR* is the natural logarithm of *RR*. It indicates a positive effect of nitrogen chemical nitrogen fertilizers on the variable if the value of ln*RR* is above 0, however, a negative effect is exhibited of nitrogen chemical nitrogen fertilizers when ln*RR* is below 0. The ln*RR* was estimated as:

3 $$ \ln RR = \ln (Mt/M{\text{c}}) = \ln Mt - \ln Mc $$

The variance (*V*) was calculated by:

4 $$ V = \frac{{SDt^{2} }}{{{\text{n}}tM{\text{t}}^{{2}} }} + \frac{{SDc^{{2}} }}{{{\text{nc}}Mc^{2} }} $$

where n_*t*_ and n_*c*_ represent the sample sizes of the treatment and control groups, respectively, and *SDt* and *SDc* represent the *SD* of the nitrogen chemical fertilizers reduction and control groups, respectively.

In addition, weighted factor (*W*_ij_), weighted response ratio (*RR*_++_), standard errors of *RR*_++_ (*S*(*RR*_++_)), and 95% confidence interval (95% *CI*) were calculated by:

5 $$ W_{{{\text{ij}}}} = 1/V $$

6 $$ RR_{ + + } = \sum\limits_{i = 1}^{m} {\sum\limits_{j = 1}^{ki} {W_{ij} } } RR_{ij} /\sum\limits_{i = 1}^{m} {\sum\limits_{j = 1}^{ki} {W_{ij} } } $$

In this paper, *RR*_++_ was described as RR_++_ × 100%.

7 $$ S(RR_{ + + } ) = 1/\sqrt {\sum\limits_{i = 1}^{m} {\sum\limits_{j = 1}^{ki} {W_{ij} } } } $$

8 $$ 95{\text{\% }}CI = RR_{ + + } \pm 1.96S(RR_{ + + } ) $$

The responses of the variable to chemical nitrogen fertilizers reduction differ significantly from control in given categories (i.e., experimental duration, reduction magnitude, reduction pattern and soil use) if the 95%*CI* value of *RR*_++_ for a given variable does not cover zero.

The frequency distributions of ln*RR* were assumed to follow normal distributions and fitted by a Gaussian function (i.e., normal distribution).

9 $$ {\text{y}} = ae^{{\frac{{\left( {x - \mu } \right)^{2} }}{{2\sigma^{2} }}}} $$

where y is the number of ln*RR* values, *a* is a coefficient showing the expected number of ln*RR* values at x = μ, and x and μ are the mean and variance of the frequency distributions of ln*RR*, respectively.

## Supplementary Information

Supplementary Information.
