# Supplementary material for: The responses of soil organic carbon and total nitrogen to chemical nitrogen fertilizers reduction base on a meta-analysis
Source: Sci Rep. 2022 Sep 29;12:16326. doi: 10.1038/s41598-022-18684-w (PMC9522798; doi:10.1038/s41598-022-18684-w)
Supplement: Supplementary file 1 — Supplementary Information. [file 41598_2022_18684_MOESM1_ESM.docx]

**The responses of soil organic carbon and total nitrogen to chemical nitrogen fertilizers reduction base on a meta-analysis**

Chuanzong Li,Oluwaseun Olayemi Aluko, Guang Yuan, Jiayi Li, Haobao Liu*

Tobacco Research Institute, Chinese Academy of Agricultural Sciences (CAAS), Qingdao, China

*Author for Correspondence: Haobao Liu

Table S1. Detailed information of experiments included in the meta-analysis.

| References | Average temperature | Average precipitation | Effective accumulated temperature | Duration years | Reduced application rate | Organic fertilizer | SOC | sd | TN | sd | SOC/TN | Sd |
| --- | --- | --- | --- | --- | --- | --- | --- | --- | --- | --- | --- | --- |
| Bei et al., 2018 ^1^ | 13.2℃ | 494 mm | 4472.0℃ | 4 | 0 | ------ | 8.31 | 1.59 | 1.01 | 0.07 | 8.23 | 0.82 |
| Bei et al., 2018 ^1^ | 13.2℃ | 494 mm | 4472.0℃ | 4 | 30 | ------ | 8.74 | 0.78 | 1.11 | 0.09 | 7.87 | 0.79 |
| Bei et al., 2018 ^1^ | 13.2℃ | 494 mm | 4472.0℃ | 4 | 30 | ------ | 10.29 | 2.22 | 1.2 | 0.14 | 8.57 | 0.86 |
| Bei et al., 2018 ^1^ | 13.2℃ | 494 mm | 4472.0℃ | 4 | 30 | ------ | 10.92 | 1.35 | 1.25 | 0 | 8.73 | 0.87 |
| Bei et al., 2018 ^1^ | 13.2℃ | 494 mm | 4472.0℃ | 4 | 0 | ------ | 8.9 | 0.33 | 0.92 | 0.16 | 9.67 | 0.97 |
| Bei et al., 2018 ^1^ | 13.2℃ | 494 mm | 4472.0℃ | 4 | 30 | ------ | 8.85 | 0.48 | 1.06 | 0.19 | 8.34 | 0.83 |
| Bei et al., 2018 ^1^ | 13.2℃ | 494 mm | 4472.0℃ | 4 | 30 | ------ | 9.52 | 1.13 | 1.16 | 0.09 | 8.21 | 0.82 |
| Bei et al., 2018 ^1^ | 13.2℃ | 494 mm | 4472.0℃ | 4 | 30 | ------ | 10.92 | 0.78 | 1.25 | 0 | 8.73 | 0.87 |
| Chen et al., 2017 ^2^ | 13.6℃ | 658.5mm | 4505℃ | 5 | 0 | ------ | 5.59 | 0.66 | 0.81 | 0.11 | 6.9 | 0.69 |
| Chen et al., 2017 ^2^ | 13.6℃ | 658.5mm | 4505℃ | 5 | 0 | ------ | 8.48 | 0.33 | 1.28 | 0.33 | 6.63 | 0.66 |
| Chen et al., 2017 ^2^ | 13.6℃ | 658.5mm | 4505℃ | 5 | 0 | ------ | 7.91 | 0.33 | 1.22 | 0.11 | 6.48 | 0.65 |
| Chen et al., 2017 ^2^ | 13.6℃ | 658.5mm | 4505℃ | 5 | 0 | ------ | 8.14 | 0.49 | 1.28 | 0.22 | 6.37 | 0.64 |
| Chen et al., 2017 ^2^ | 13.6℃ | 658.5mm | 4505℃ | 5 | 30 | ------ | 5.24 | 0.16 | 0.69 | 0.07 | 7.63 | 0.76 |
| Chen et al., 2017 ^2^ | 13.6℃ | 658.5mm | 4505℃ | 5 | 30 | ------ | 8.37 | 0.33 | 1.17 | 0.16 | 7.16 | 0.72 |
| Chen et al., 2017 ^2^ | 13.6℃ | 658.5mm | 4505℃ | 5 | 30 | ------ | 7.71 | 0.49 | 1.16 | 0.16 | 6.63 | 0.66 |
| Chen et al., 2017 ^2^ | 13.6℃ | 658.5mm | 4505℃ | 5 | 30 | ------ | 7.94 | 0.33 | 1.18 | 0.13 | 6.75 | 0.68 |
| Chen et al., 2020 ^3^ | 16.6℃ | 1315 mm | 4753.6℃ | 37 | 100 | ------ | 15.24 | 0.48 | 0.83 | 0.1 | 18.36 | 1.84 |
| Chen et al., 2020 ^3^ | 16.6℃ | 1315 mm | 4753.6℃ | 37 | 50 | soybean cake | 23 | 0.61 | 1.19 | 0.02 | 19.33 | 1.93 |
| Chen et al., 2020 ^3^ | 16.6℃ | 1315 mm | 4753.6℃ | 37 | 0 | ------ | 17.28 | 0.23 | 1.05 | 0.21 | 16.46 | 1.65 |
| Cheng et al., 2014 ^4^ | 15.7℃ | 940mm | 5100℃ | 1 | 100 | ------ | 13.36 | 1.34 | 1.47 | 0.15 | 9.09 | 0.91 |
| Cheng et al., 2014 ^4^ | 15.7℃ | 940mm | 5100℃ | 1 | 50 | ------ | 15.66 | 1.57 | 1.82 | 0.18 | 8.6 | 0.86 |
| Cheng et al., 2014 ^4^ | 15.7℃ | 940mm | 5100℃ | 1 | 33 | ------ | 18.08 | 1.81 | 1.9 | 0.19 | 9.52 | 0.95 |
| Cheng et al., 2014 ^4^ | 15.7℃ | 940mm | 5100℃ | 1 | 16 | ------ | 18.08 | 1.81 | 2.07 | 0.21 | 8.73 | 0.87 |
| Cheng et al., 2014 ^4^ | 15.7℃ | 940mm | 5100℃ | 1 | 0 | ------ | 19.77 | 1.98 | 2.11 | 0.21 | 9.37 | 0.94 |
| He et al., 2019 ^5^ | 16.5℃ | 1550 mm | 6000℃ | 2 | 0 | ------ | 17.57 | 2.08 | 2.49 | 0.17 | 7.06 | 0.71 |
| He et al., 2019 ^5^ | 16.6℃ | 1551 mm | 6001℃ | 2 | 30 | ------ | 21.34 | 1.04 | 2.5 | 0.16 | 8.54 | 0.85 |
| Hou et al., 2012 ^6^ | 17.5℃ | 1600 mm | 5400℃ | 2 | 100 | ------ | 9.15 | 0.92 | 1.36 | 0.14 | 6.73 | 0.67 |
| Hou et al., 2012 ^6^ | 17.5℃ | 1600 mm | 5400℃ | 2 | 0 | ------ | 15.96 | 1.6 | 1.49 | 0.15 | 10.71 | 1.07 |
| Hou et al., 2012 ^6^ | 17.5℃ | 1600 mm | 5400℃ | 2 | 20 | Organic fertilizer | 16.86 | 1.69 | 1.47 | 0.15 | 11.47 | 1.15 |
| Hou et al., 2012 ^6^ | 17.5℃ | 1600 mm | 5400℃ | 2 | 20 | ------ | 15.8 | 1.58 | 1.65 | 0.17 | 9.58 | 0.96 |
| Hou et al., 2012 ^6^ | 17.5℃ | 1600 mm | 5400℃ | 2 | 70 | Organic fertilizer | 17.87 | 1.79 | 1.71 | 0.17 | 10.45 | 1.05 |
| Hou et al., 2012 ^6^ | 17.5℃ | 1600 mm | 5400℃ | 2 | 70 | Organic fertilizer | 17.54 | 1.75 | 1.63 | 0.16 | 10.76 | 1.08 |
| Hou et al., 2012 ^6^ | 17.5℃ | 1600 mm | 5400℃ | 2 | 50 | Organic fertilizer | 16.33 | 1.63 | 1.55 | 0.16 | 10.53 | 1.05 |
| Hou et al., 2012 ^6^ | 17.5℃ | 1600 mm | 5400℃ | 2 | 20 | ------ | 16.12 | 1.61 | 1.6 | 0.16 | 10.08 | 1.01 |
| Hou et al., 2012 ^6^ | 17.5℃ | 1600 mm | 5400℃ | 3 | 100 | ------ | 15 | 1.5 | 1.13 | 0.11 | 13.27 | 1.33 |
| Hou et al., 2012 ^6^ | 17.6℃ | 1601 mm | 5401℃ | 3 | 0 | ------ | 14.96 | 1.5 | 1.29 | 0.13 | 11.6 | 1.16 |
| Hou et al., 2012 ^6^ | 17.7℃ | 1602 mm | 5402℃ | 3 | 20 | Organic fertilizer | 14.24 | 1.42 | 1.25 | 0.13 | 11.39 | 1.14 |
| Hou et al., 2012 ^6^ | 17.8℃ | 1603 mm | 5403℃ | 3 | 20 | ------ | 13.17 | 1.32 | 1.26 | 0.13 | 10.45 | 1.04 |
| Hou et al., 2012 ^6^ | 17.9℃ | 1604 mm | 5404℃ | 3 | 70 | Organic fertilizer | 17.28 | 1.73 | 1.33 | 0.13 | 13 | 1.3 |
| Hou et al., 2012 ^6^ | 17.10℃ | 1605 mm | 5405℃ | 3 | 70 | Organic fertilizer | 15.53 | 1.55 | 1.26 | 0.13 | 12.33 | 1.23 |
| Hou et al., 2012 ^6^ | 17.11℃ | 1606 mm | 5406℃ | 3 | 50 | Organic fertilizer | 15.52 | 1.55 | 1.29 | 0.13 | 12.03 | 1.2 |
| Hou et al., 2012 ^6^ | 17.12℃ | 1607 mm | 5407℃ | 3 | 20 | ------ | 13.91 | 1.39 | 1.14 | 0.11 | 12.2 | 1.22 |
| Lan et al., 2017 ^7^ | 6.4℃ | 390.9mm | 2239.1℃ | 8 | 100 | ------ | 8.02 | 0.6 | 1.02 | 0.2 | 7.83 | 0.78 |
| Lan et al., 2017 ^7^ | 6.4℃ | 390.9mm | 2239.1℃ | 8 | 50 | ------ | 8.42 | 2.4 | 1.06 | 0.1 | 7.96 | 0.8 |
| Lan et al., 2017 ^7^ | 6.4℃ | 390.9mm | 2239.1℃ | 8 | 0 | ------ | 9.4 | 1.4 | 1.07 | 0.12 | 8.78 | 0.88 |
| Li et al., 2012 ^8^ | 18.2℃ | 1700 mm | 5860℃ | 1 | 100 | ------ | 15.52 | 0.66 | 1.06 | 0.03 | 14.64 | 1.46 |
| Li et al., 2012 ^8^ | 18.2℃ | 1700 mm | 5860℃ | 1 | 0 | ------ | 11.3 | 0.05 | 1.28 | 0.02 | 8.83 | 0.88 |
| Li et al., 2012 ^8^ | 18.2℃ | 1700 mm | 5860℃ | 1 | 20 | ------ | 11.78 | 0.94 | 1.36 | 0.02 | 8.66 | 0.87 |
| Li et al., 2017 ^9^ | 8℃ | 213mm | 3463.5 ℃ | 4 | 100 | ------ | 12.9 | 1.29 | 0.11 | 0.01 | 117.27 | 11.73 |
| Li et al., 2017 ^9^ | 8℃ | 213mm | 3463.5 ℃ | 4 | 0 | ------ | 14.8 | 1.48 | 0.14 | 0.01 | 105.71 | 10.57 |
| Li et al., 2017 ^9^ | 8℃ | 213mm | 3463.5 ℃ | 4 | 20 | Biological fertilizer | 16 | 1.6 | 0.16 | 0.02 | 100 | 10 |
| Li et al., 2017 ^9^ | 8℃ | 213mm | 3463.5 ℃ | 4 | 40 | Biological fertilizer | 17.1 | 1.71 | 0.15 | 0.02 | 114 | 11.4 |
| Li et al., 2017 ^9^ | 8℃ | 213mm | 3463.5 ℃ | 4 | 20 | Organic fertilizer | 16.6 | 1.66 | 0.15 | 0.02 | 110.67 | 11.07 |
| Li et al., 2017 ^9^ | 8℃ | 213mm | 3463.5 ℃ | 4 | 40 | Organic fertilizer | 18.1 | 1.81 | 0.18 | 0.02 | 100.56 | 10.06 |
| Liang et al., 2019 ^10^ | 12. 9 ℃ | 550 mm | 3650℃ | 3 | 100 | ------ | 8.76 | 0.88 | 0.18 | 0.02 | 48.09 | 4.81 |
| Liang et al., 2019 ^10^ | 12. 9 ℃ | 550 mm | 3650℃ | 3 | 75 | ------- | 7.97 | 0.8 | 0.56 | 0.06 | 14.16 | 1.42 |
| Liang et al., 2019 ^10^ | 12. 9 ℃ | 550 mm | 3650℃ | 3 | 50 | ----- | 8.57 | 0.86 | 0.4 | 0.04 | 21.62 | 2.16 |
| Liang et al., 2019 ^10^ | 12. 9 ℃ | 550 mm | 3650℃ | 3 | 25 | ------ | 8.86 | 0.89 | 0.4 | 0.04 | 22.37 | 2.24 |
| Liang et al., 2019 ^10^ | 12. 9 ℃ | 550 mm | 3650℃ | 3 | 0 | ------ | 8.76 | 0.88 | 0.37 | 0.04 | 23.53 | 2.35 |
| Liang et al., 2019 ^10^ | 12. 9 ℃ | 550 mm | 3650℃ | 3 | 100 | Cow dung | 10.56 | 1.06 | 0.84 | 0.08 | 12.57 | 1.26 |
| Liang et al., 2019 ^10^ | 12. 9 ℃ | 550 mm | 3650℃ | 3 | 75 | ----- | 11.45 | 1.15 | 1.04 | 0.1 | 11.03 | 1.1 |
| Liang et al., 2019 ^10^ | 12. 9 ℃ | 550 mm | 3650℃ | 3 | 50 | -------- | 11.85 | 1.19 | 1.05 | 0.1 | 11.33 | 1.13 |
| Liang et al., 2019 ^10^ | 12. 9 ℃ | 550 mm | 3650℃ | 3 | 25 | ----- | 11.35 | 1.14 | 0.95 | 0.1 | 11.94 | 1.19 |
| Liang et al., 2019 ^10^ | 12. 9 ℃ | 550 mm | 3650℃ | 3 | 0 | ------ | 11.35 | 1.14 | 0.99 | 0.1 | 11.46 | 1.15 |
| Liang et al., 2019 ^10^ | 12. 9 ℃ | 550 mm | 3650℃ | 4 | 100 | ------- | 6.73 | 0.67 | 0.38 | 0.04 | 17.85 | 1.79 |
| Liang et al., 2019 ^10^ | 12. 9 ℃ | 550 mm | 3650℃ | 4 | 75 | ------ | 8.01 | 0.8 | 0.39 | 0.04 | 20.44 | 2.04 |
| Liang et al., 2019 ^10^ | 12. 9 ℃ | 550 mm | 3650℃ | 4 | 50 | ------ | 9.61 | 0.96 | 0.39 | 0.04 | 24.52 | 2.45 |
| Liang et al., 2019 ^10^ | 12. 9 ℃ | 550 mm | 3650℃ | 4 | 25 | -------- | 9.82 | 0.98 | 0.44 | 0.04 | 22.5 | 2.25 |
| Liang et al., 2019 ^10^ | 12. 9 ℃ | 550 mm | 3650℃ | 4 | 0 | ------- | 9.61 | 0.96 | 0.45 | 0.05 | 21.28 | 2.13 |
| Liang et al., 2019 ^10^ | 12. 9 ℃ | 550 mm | 3650℃ | 4 | 100 | Cow dung | 13.88 | 1.39 | 1.12 | 0.11 | 12.39 | 1.24 |
| Liang et al., 2019 ^10^ | 12. 9 ℃ | 550 mm | 3650℃ | 4 | 75 | ------ | 14.2 | 1.42 | 1.11 | 0.11 | 12.76 | 1.28 |
| Liang et al., 2019 ^10^ | 12. 9 ℃ | 550 mm | 3650℃ | 4 | 50 | ----- | 17.83 | 1.78 | 1.19 | 0.12 | 15.02 | 1.5 |
| Liang et al., 2019 ^10^ | 12. 9 ℃ | 550 mm | 3650℃ | 4 | 25 | ------ | 13.35 | 1.33 | 1.15 | 0.11 | 11.61 | 1.16 |
| Liang et al., 2019 ^10^ | 12. 9 ℃ | 550 mm | 3650℃ | 4 | 0 | ------ | 15.27 | 1.53 | 1.14 | 0.11 | 13.36 | 1.34 |
| Liu et al., 2013 ^11^ | 17°C | 1549mm | 3400℃ | 1 | 100 | ------ | 7.83 | 0.02 | 0.94 | 1.23 | 8.33 | 0.83 |
| Liu et al., 2013 ^11^ | 17°C | 1550mm | 3400℃ | 1 | 75 | ------ | 8.25 | 0.01 | 0.97 | 0.03 | 8.51 | 0.85 |
| Liu et al., 2013 ^11^ | 17°C | 1551mm | 3400℃ | 1 | 25 | ------ | 8.56 | 0.02 | 1.02 | 0.12 | 8.39 | 0.84 |
| Liu et al., 2013 ^11^ | 17°C | 1552mm | 3400℃ | 1 | 0 | ------ | 8.54 | 0.04 | 1.12 | 1.21 | 7.63 | 0.76 |
| Liu et al., 2013 ^11^ | 17°C | 1553mm | 3400℃ | 1 | 50 | biochar | 7.56 | 1.04 | 1.07 | 0.21 | 7.07 | 0.71 |
| Liu et al., 2013 ^11^ | 17°C | 1554mm | 3400℃ | 1 | 25 | ------ | 7.5 | 0.83 | 1.03 | 0.74 | 7.28 | 0.73 |
| Liu et al., 2013 ^11^ | 17°C | 1555mm | 3400℃ | 1 | 0 | ------ | 7.88 | 1.23 | 1.08 | 1.02 | 7.3 | 0.73 |
| Liu et al., 2013 ^11^ | 17°C | 1556mm | 3400℃ | 1 | 50 | biochar | 7.52 | 2.34 | 1.1 | 1.23 | 6.84 | 0.68 |
| Liu et al., 2013 ^11^ | 17°C | 1557mm | 3400℃ | 1 | 25 | ------ | 7.5 | 3.45 | 1.04 | 1.43 | 7.21 | 0.72 |
| Liu et al., 2013 ^11^ | 17°C | 1558mm | 3400℃ | 1 | 0 | ------ | 8.05 | 3.42 | 1.02 | 0.82 | 7.89 | 0.79 |
| Liu et al., 2013 ^11^ | 17°C | 1559mm | 3400℃ | 1 | 50 | biochar | 8.6 | 1.87 | 1.14 | 0.54 | 7.54 | 0.75 |
| Liu et al., 2013 ^11^ | 17°C | 1560mm | 3400℃ | 1 | 25 | ------ | 12.37 | 3.87 | 1.15 | 0.93 | 10.76 | 1.08 |
| Liu et al., 2013 ^11^ | 17°C | 1561mm | 3400℃ | 1 | 0 | ------ | 9.94 | 1.56 | 1.23 | 2.34 | 8.08 | 0.81 |
| Liu et al., 2013 ^11^ | 17°C | 1562mm | 3400℃ | 1 | 50 | biochar | 9.87 | 2.56 | 1.32 | 2.12 | 7.48 | 0.75 |
| Liu et al., 2013 ^11^ | 17°C | 1563mm | 3400℃ | 1 | 25 | ------ | 9.5 | 3.01 | 1.12 | 2.43 | 8.48 | 0.85 |
| Liu et al., 2013 ^11^ | 17°C | 1564mm | 3400℃ | 1 | 0 | ------ | 11.12 | 3.57 | 1.11 | 1.21 | 10.02 | 1 |
| Liu et al., 2013 ^11^ | 17°C | 1565mm | 3400℃ | 1 | 50 | biochar | 11.44 | 2.45 | 1.32 | 2.67 | 8.67 | 0.87 |
| Liu et al., 2013 ^11^ | 17°C | 1566mm | 3400℃ | 1 | 25 | ------ | 15.2 | 5.21 | 1.35 | 2.83 | 11.26 | 1.13 |
| Liu et al., 2013 ^11^ | 17°C | 1567mm | 3400℃ | 1 | 0 | ------ | 12.47 | 4.23 | 1.24 | 1.83 | 10.06 | 1.01 |
| Liu et al., 2013 ^11^ | 17°C | 1568mm | 3400℃ | 1 | 50 | Biochar | 18.53 | 7.32 | 1.21 | 1.42 | 15.31 | 1.53 |
| Liu et al., 2013 ^11^ | 17°C | 1569mm | 3400℃ | 1 | 25 | ------ | 18.05 | 8.01 | 1.32 | 3.52 | 13.67 | 1.37 |
| Liu et al., 2013 ^11^ | 17°C | 1570mm | 3400℃ | 1 | 0 | ------ | 16.42 | 6.34 | 1.42 | 5.21 | 11.56 | 1.16 |
| Liu et al., 2016 ^12^ | 15.2℃ | 1058.8mm | 4859.6℃ | 1 | 100 | ------ | 6.45 | 0.02 | 1.25 | 0.1 | 5.15 | 0.52 |
| Liu et al., 2016 ^12^ | 15.2℃ | 1058.8mm | 4859.6℃ | 1 | 66 | ------ | 6.95 | 0.08 | 1.29 | 0.14 | 5.37 | 0.54 |
| Liu et al., 2016 ^12^ | 15.2℃ | 1058.8mm | 4859.6℃ | 1 | 33 | ------ | 6.97 | 0.05 | 1.33 | 0.18 | 5.24 | 0.52 |
| Liu et al., 2016 ^12^ | 15.2℃ | 1058.8mm | 4859.6℃ | 1 | 0 | ------ | 7.05 | 0.13 | 1.35 | 0.2 | 5.22 | 0.52 |
| Liu et al., 2020 ^13^ | 13.37 ℃ | 640 mm | 4 485.1℃ | 10 | 100 | -------- | 9.38 | 0.94 | 0.95 | 0.1 | 9.87 | 0.99 |
| Liu et al., 2020 ^13^ | 13.37 ℃ | 640 mm | 4 485.1℃ | 10 | 75 | -------- | 9.66 | 0.97 | 1.02 | 0.1 | 9.47 | 0.95 |
| Liu et al., 2020 ^13^ | 13.37 ℃ | 640 mm | 4 485.1℃ | 10 | 50 | -------- | 10.19 | 1.02 | 1.05 | 0.11 | 9.7 | 0.97 |
| Liu et al., 2020 ^13^ | 13.37 ℃ | 640 mm | 4 485.1℃ | 10 | 25 | -------- | 10.53 | 1.05 | 1.07 | 0.11 | 9.84 | 0.98 |
| Liu et al., 2020 ^13^ | 13.37 ℃ | 640 mm | 4 485.1℃ | 10 | 0 | -------- | 10.36 | 1.04 | 1.1 | 0.11 | 9.42 | 0.94 |
| Liu et al., 2020 ^13^ | 13.37 ℃ | 640 mm | 4 485.1℃ | 10 | 100 | -------- | 9.2 | 0.92 | 0.99 | 0.1 | 9.29 | 0.93 |
| Liu et al., 2020 ^13^ | 13.37 ℃ | 640 mm | 4 485.1℃ | 10 | 75 | -------- | 9.89 | 0.99 | 1.03 | 0.1 | 9.6 | 0.96 |
| Liu et al., 2020 ^13^ | 13.37 ℃ | 640 mm | 4 485.1℃ | 10 | 50 | -------- | 10.51 | 1.05 | 1.09 | 0.11 | 9.64 | 0.96 |
| Liu et al., 2020 ^13^ | 13.37 ℃ | 640 mm | 4 485.1℃ | 10 | 25 | -------- | 10.47 | 1.05 | 1.12 | 0.11 | 9.35 | 0.93 |
| Liu et al., 2020 ^13^ | 13.37 ℃ | 640 mm | 4 485.1℃ | 10 | 0 | -------- | 10.34 | 1.03 | 1.18 | 0.12 | 8.76 | 0.88 |
| Lu et al., 2015 ^14^ | 7.8℃ | 158 mm | 3200℃ | 3 | 100 | ------ | 11.96 | 1.2 | 1.23 | 0.12 | 9.75 | 0.98 |
| Lu et al., 2015 ^14^ | 7.8℃ | 158 mm | 3200℃ | 3 | 0 | ------ | 12.43 | 1.24 | 1.29 | 0.13 | 9.62 | 0.96 |
| Lu et al., 2015 ^14^ | 7.8℃ | 158 mm | 3200℃ | 3 | 5 | Green manure | 12.52 | 1.25 | 1.29 | 0.13 | 9.72 | 0.97 |
| Lu et al., 2015 ^14^ | 7.8℃ | 158 mm | 3200℃ | 3 | 10 | Green manure | 12.31 | 1.23 | 1.29 | 0.13 | 9.52 | 0.95 |
| Lu et al., 2015 ^14^ | 7.8℃ | 158 mm | 3200℃ | 3 | 15 | Green manure | 12.17 | 1.22 | 1.27 | 0.13 | 9.61 | 0.96 |
| Lu et al., 2015 ^14^ | 7.8℃ | 158 mm | 3200℃ | 3 | 20 | Green manure | 12.19 | 1.22 | 1.24 | 0.12 | 9.83 | 0.98 |
| Lu et al., 2015 ^14^ | 7.8℃ | 158 mm | 3200℃ | 3 | 100 | Green manure | 12.12 | 1.21 | 1.23 | 0.12 | 9.86 | 0.99 |
| Lu et al., 2015 ^14^ | 7.8℃ | 158 mm | 3200℃ | 3 | 10 | Green manure | 12.5 | 1.25 | 1.23 | 0.12 | 10.2 | 1.02 |
| Lu et al., 2015 ^14^ | 7.8℃ | 158 mm | 3200℃ | 3 | 20 | Green manure | 12.39 | 1.24 | 1.29 | 0.13 | 9.59 | 0.96 |
| Lu et al., 2015 ^14^ | 7.8℃ | 158 mm | 3200℃ | 3 | 30 | Green manure | 12.33 | 1.23 | 1.31 | 0.13 | 9.44 | 0.94 |
| Lu et al., 2015 ^14^ | 7.8℃ | 158 mm | 3200℃ | 3 | 40 | Green manure | 12.21 | 1.22 | 1.3 | 0.13 | 9.38 | 0.94 |
| Lu et al., 2015 ^14^ | 7.8℃ | 158 mm | 3200℃ | 3 | 100 | Green manure | 12.17 | 1.22 | 1.28 | 0.13 | 9.5 | 0.95 |
| Ma et al., 2015 ^15^ | 14.6°C | 557.2mm | 4700℃ | 1 | 0 | ------ | 18.17 | 1.26 | 1.07 | 0.14 | 16.92 | 1.69 |
| Ma et al., 2015 ^15^ | 14.6°C | 557.2mm | 4700℃ | 1 | 10 | straw | 13 | 1.26 | 0.8 | 0.09 | 16.21 | 1.62 |
| Ma et al., 2015 ^15^ | 14.6°C | 557.2mm | 4700℃ | 1 | 15 | Cow dung | 14.55 | 1.57 | 0.98 | 0.02 | 14.82 | 1.48 |
| Ma et al., 2015 ^15^ | 14.6°C | 557.2mm | 4700℃ | 1 | 15 | Biogas slurry | 12.45 | 0.94 | 0.76 | 0.28 | 16.34 | 1.63 |
| Ma et al., 2018 ^16^ | 16.5℃ | 1389mm | 6539°C | 4 | 100 | - | 16.43 | 2.42 | 1.53 | 0.19 | 10.71 | 1.07 |
| Ma et al., 2018 ^16^ | 16.5℃ | 1389mm | 6539°C | 4 | 0 | - | 18.01 | 2.42 | 1.56 | 0.45 | 11.56 | 1.16 |
| Ma et al., 2018 ^16^ | 16.5℃ | 1389mm | 6539°C | 4 | 25 | - | 17.74 | 0.91 | 1.62 | 0.21 | 10.96 | 1.1 |
| Ma et al., 2018 ^16^ | 16.5℃ | 1389mm | 6539°C | 4 | 50 | straw | 21.07 | 1.21 | 1.6 | 0.61 | 13.2 | 1.32 |
| Ma et al., 2018 ^16^ | 16.5℃ | 1389mm | 6539°C | 4 | 50 | cow dung | 19.41 | 1.21 | 1.64 | 0.69 | 11.82 | 1.18 |
| Ma et al., 2018 ^16^ | 16.5℃ | 1389mm | 6539°C | 4 | 75 | cow dung | 18.44 | 1.82 | 1.63 | 0.37 | 11.29 | 1.13 |
| Ma et al., 2018 ^16^ | 12.9℃ | 550mm | 3650℃ | 3 | 100 | ------ | 7.83 | 0.78 | 0.81 | 0.08 | 9.67 | 0.97 |
| Ma et al., 2018 ^16^ | 12.10℃ | 551mm | 3650℃ | 3 | 75 | ------ | 8.18 | 0.82 | 0.73 | 0.07 | 11.2 | 1.12 |
| Ma et al., 2018 ^16^ | 12.10℃ | 552mm | 3650℃ | 3 | 50 | ------ | 8.76 | 0.88 | 0.84 | 0.08 | 10.43 | 1.04 |
| Ma et al., 2018 ^16^ | 12.10℃ | 553mm | 3650℃ | 3 | 25 | ------ | 8.58 | 0.86 | 0.81 | 0.08 | 10.6 | 1.06 |
| Ma et al., 2018 ^16^ | 12.10℃ | 554mm | 3650℃ | 3 | 0 | ------ | 7.89 | 0.79 | 0.81 | 0.08 | 9.74 | 0.97 |
| Ma et al., 2018 ^16^ | 12.10℃ | 555mm | 3650℃ | 3 | 100 | Cow dung | 10.15 | 1.02 | 0.86 | 0.09 | 11.8 | 1.18 |
| Ma et al., 2018 ^16^ | 12.10℃ | 556mm | 3650℃ | 3 | 75 | ------ | 10.27 | 1.03 | 0.86 | 0.09 | 11.94 | 1.19 |
| Ma et al., 2018 ^16^ | 12.10℃ | 557mm | 3650℃ | 3 | 50 | ------ | 10.38 | 1.04 | 0.94 | 0.09 | 11.04 | 1.1 |
| Ma et al., 2018 ^16^ | 12.10℃ | 558mm | 3650℃ | 3 | 25 | ------ | 10.61 | 1.06 | 0.82 | 0.08 | 12.94 | 1.29 |
| Ma et al., 2018 ^16^ | 12.10℃ | 559mm | 3650℃ | 3 | 0 | ------ | 10.21 | 1.02 | 0.88 | 0.09 | 11.6 | 1.16 |
| Meng et al., 2018 ^17^ | 6.6℃ | 350 mm | 3200℃ | 1 | 100 | ------ | 11.55 | 1.16 | 1.16 | 0.12 | 9.96 | 1 |
| Meng et al., 2018 ^17^ | 6.6℃ | 350 mm | 3200℃ | 1 | 50 | ------ | 11.64 | 1.16 | 1 | 0.1 | 11.64 | 1.16 |
| Meng et al., 2018 ^17^ | 6.6℃ | 350 mm | 3200℃ | 1 | 0 | ------ | 11.54 | 1.15 | 1.02 | 0.1 | 11.31 | 1.13 |
| Meng et al., 2018 ^17^ | 6.6℃ | 350 mm | 3200℃ | 1 | 100 | biochar | 12.75 | 1.28 | 1.07 | 0.11 | 11.92 | 1.19 |
| Meng et al., 2018 ^17^ | 6.6℃ | 350 mm | 3200℃ | 1 | 50 | ------ | 12.72 | 1.27 | 1.08 | 0.11 | 11.78 | 1.18 |
| Meng et al., 2018 ^17^ | 6.6℃ | 350 mm | 3200℃ | 1 | 0 | ------ | 13.03 | 1.3 | 1.11 | 0.11 | 11.74 | 1.17 |
| Meng et al., 2018 ^17^ | 6.6℃ | 350 mm | 3200℃ | 1 | 100 | biochar | 13.89 | 1.39 | 1.12 | 0.11 | 12.4 | 1.24 |
| Meng et al., 2018 ^17^ | 6.6℃ | 350 mm | 3200℃ | 1 | 50 | ------ | 13.93 | 1.39 | 1.14 | 0.11 | 12.22 | 1.22 |
| Meng et al., 2018 ^17^ | 6.6℃ | 350 mm | 3200℃ | 1 | 0 | ------ | 14.59 | 1.46 | 1.15 | 0.12 | 12.69 | 1.27 |
| Meng et al., 2018 ^17^ | 6.6℃ | 350 mm | 3200℃ | 1 | 100 | biochar | 14.47 | 1.45 | 1.2 | 0.12 | 12.06 | 1.21 |
| Meng et al., 2018 ^17^ | 6.6℃ | 350 mm | 3200℃ | 1 | 50 | ------ | 14.87 | 1.49 | 1.21 | 0.12 | 12.29 | 1.23 |
| Meng et al., 2018 ^17^ | 6.6℃ | 350 mm | 3200℃ | 1 | 0 | ------ | 15.13 | 1.51 | 1.22 | 0.12 | 12.4 | 1.24 |
| Meng et al., 2018 ^17^ | 8.5℃ | 262.9mm | 2965℃ | 1 | 100 | ------ | 11.51 | 1.15 | 1.05 | 0.11 | 10.96 | 1.1 |
| Meng et al., 2018 ^17^ | 8.5℃ | 262.9mm | 2965℃ | 1 | 50 | ------ | 12.02 | 1.2 | 1.06 | 0.11 | 11.34 | 1.13 |
| Meng et al., 2018 ^17^ | 8.5℃ | 262.9mm | 2965℃ | 1 | 0 | ------ | 12.23 | 1.22 | 1.06 | 0.11 | 11.54 | 1.15 |
| Meng et al., 2018 ^17^ | 8.5℃ | 262.9mm | 2965℃ | 1 | 100 | biochar | 12.64 | 1.26 | 1.05 | 0.11 | 12.04 | 1.2 |
| Meng et al., 2018 ^17^ | 8.5℃ | 262.9mm | 2965℃ | 1 | 50 | ------ | 13.04 | 1.3 | 1.06 | 0.11 | 12.3 | 1.23 |
| Meng et al., 2018 ^17^ | 8.5℃ | 262.9mm | 2965℃ | 1 | 0 | ------ | 13.04 | 1.3 | 1.07 | 0.11 | 12.19 | 1.22 |
| Meng et al., 2018 ^17^ | 8.5℃ | 262.9mm | 2965℃ | 1 | 100 | biochar | 13.45 | 1.35 | 1.13 | 0.11 | 11.9 | 1.19 |
| Meng et al., 2018 ^17^ | 8.5℃ | 262.9mm | 2965℃ | 1 | 50 | ------ | 13.9 | 1.39 | 1.15 | 0.12 | 12.09 | 1.21 |
| Meng et al., 2018 ^17^ | 8.5℃ | 262.9mm | 2965℃ | 1 | 0 | ------ | 14.64 | 1.46 | 1.18 | 0.12 | 12.41 | 1.24 |
| Meng et al., 2018 ^17^ | 8.5℃ | 262.9mm | 2965℃ | 1 | 100 | biochar | 14.68 | 1.47 | 1.19 | 0.12 | 12.34 | 1.23 |
| Meng et al., 2018 ^17^ | 8.5℃ | 262.9mm | 2965℃ | 1 | 50 | ------ | 15.06 | 1.51 | 1.2 | 0.12 | 12.55 | 1.26 |
| Meng et al., 2018 ^17^ | 8.5℃ | 262.9mm | 2965℃ | 1 | 0 | ------ | 15.33 | 1.53 | 1.21 | 0.12 | 12.67 | 1.27 |
| Song et al., 2017 ^18^ | 14.4℃ | 640.9mm | 4925℃ | 1 | 100 | ------ | 4.23 | 0.42 | 0.52 | 0.05 | 8.13 | 0.81 |
| Song et al., 2017 ^18^ | 14.4℃ | 640.9mm | 4925℃ | 1 | 50 | ------ | 4.43 | 0.44 | 0.54 | 0.05 | 8.2 | 0.82 |
| Song et al., 2017 ^18^ | 14.4℃ | 640.9mm | 4925℃ | 1 | 25 | ------ | 4.29 | 0.43 | 0.55 | 0.06 | 7.8 | 0.78 |
| Song et al., 2017 ^18^ | 14.4℃ | 640.9mm | 4925℃ | 1 | 0 | ------ | 4.29 | 0.43 | 0.56 | 0.06 | 7.66 | 0.77 |
| Song et al., 2017 ^18^ | 14.4℃ | 640.9mm | 4925℃ | 1 | 100 | ------ | 4.31 | 0.43 | 0.48 | 0.05 | 8.98 | 0.9 |
| Song et al., 2017 ^18^ | 14.4℃ | 640.9mm | 4925℃ | 1 | 50 | ------ | 4.78 | 0.48 | 0.53 | 0.05 | 9.02 | 0.9 |
| Song et al., 2017 ^18^ | 14.4℃ | 640.9mm | 4925℃ | 1 | 25 | ------ | 4.25 | 0.43 | 0.52 | 0.05 | 8.17 | 0.82 |
| Song et al., 2017 ^18^ | 14.4℃ | 640.9mm | 4925℃ | 1 | 0 | ------ | 4.52 | 0.45 | 0.57 | 0.06 | 7.93 | 0.79 |
| Song et al., 2017 ^18^ | 14.4℃ | 640.9mm | 4925℃ | 1 | 100 | biochar | 5.96 | 0.6 | 0.67 | 0.07 | 8.9 | 0.89 |
| Song et al., 2017 ^18^ | 14.4℃ | 640.9mm | 4925℃ | 1 | 50 | ------ | 6.21 | 0.62 | 0.68 | 0.07 | 9.13 | 0.91 |
| Song et al., 2017 ^18^ | 14.4℃ | 640.9mm | 4925℃ | 1 | 25 | ------ | 6.12 | 0.61 | 0.7 | 0.07 | 8.74 | 0.87 |
| Song et al., 2017 ^18^ | 14.4℃ | 640.9mm | 4925℃ | 1 | 0 | ------ | 5.92 | 0.59 | 0.78 | 0.08 | 7.59 | 0.76 |
| Song et al., 2017 ^18^ | 14.4℃ | 640.9mm | 4925℃ | 1 | 100 | BC7.5 生物炭 | 6.5 | 0.65 | 0.63 | 0.06 | 10.32 | 1.03 |
| Song et al., 2017 ^18^ | 14.4℃ | 640.9mm | 4925℃ | 1 | 50 | ------ | 6.72 | 0.67 | 0.64 | 0.06 | 10.5 | 1.05 |
| Song et al., 2017 ^18^ | 14.4℃ | 640.9mm | 4925℃ | 1 | 25 | ------ | 6.81 | 0.68 | 0.66 | 0.07 | 10.32 | 1.03 |
| Song et al., 2017 ^18^ | 14.4℃ | 640.9mm | 4925℃ | 1 | 0 | ------ | 6.06 | 0.61 | 0.67 | 0.07 | 9.04 | 0.9 |
| Song et al., 2017 ^18^ | 14.4℃ | 640.9mm | 4925℃ | 1 | 100 | BC22.5 生物炭 | 8.51 | 0.85 | 0.79 | 0.08 | 10.77 | 1.08 |
| Song et al., 2017 ^18^ | 14.4℃ | 640.9mm | 4925℃ | 1 | 50 | ------ | 9.53 | 0.95 | 0.82 | 0.08 | 11.62 | 1.16 |
| Song et al., 2017 ^18^ | 14.4℃ | 640.9mm | 4925℃ | 1 | 25 | ------ | 8.95 | 0.9 | 0.86 | 0.09 | 10.41 | 1.04 |
| Song et al., 2017 ^18^ | 14.4℃ | 640.9mm | 4925℃ | 1 | 0 | ------ | 8.8 | 0.88 | 0.88 | 0.09 | 10 | 1 |
| Song et al., 2017 ^18^ | 14.4℃ | 640.9mm | 4925℃ | 1 | 100 | BC22.5 生物炭 | 9.12 | 0.91 | 0.76 | 0.08 | 12 | 1.2 |
| Song et al., 2017 ^18^ | 14.4℃ | 640.9mm | 4925℃ | 1 | 50 | ------ | 11.83 | 1.18 | 0.87 | 0.09 | 13.6 | 1.36 |
| Song et al., 2017 ^18^ | 14.4℃ | 640.9mm | 4925℃ | 1 | 25 | ------ | 11.26 | 1.13 | 0.9 | 0.09 | 12.51 | 1.25 |
| Song et al., 2017 ^18^ | 14.4℃ | 640.9mm | 4925℃ | 1 | 0 | ------ | 8.34 | 0.83 | 0.92 | 0.09 | 9.07 | 0.91 |
| Tao et al., 2017 ^19^ | 15.7℃ | 1100mm | 5750℃ | 4 | 0 | ------ | 18.35 | 1.83 | 1.92 | 0.19 | 9.54 | 0.95 |
| Tao et al., 2017 ^19^ | 15.7℃ | 1100mm | 5750℃ | 4 | 20 | Green manure | 20.69 | 2.07 | 2.1 | 0.21 | 9.86 | 0.99 |
| Tao et al., 2017 ^19^ | 15.7℃ | 1100mm | 5750℃ | 4 | 40 | Green manure | 21.31 | 2.13 | 2.21 | 0.22 | 9.66 | 0.97 |
| Tao et al., 2017 ^19^ | 15.7℃ | 1100mm | 5750℃ | 4 | 60 | Green manure | 22.05 | 2.2 | 2.34 | 0.23 | 9.42 | 0.94 |
| Tao et al., 2017 ^19^ | 15.7℃ | 1100mm | 5750℃ | 4 | 80 | Green manure | 22.88 | 2.29 | 2.35 | 0.24 | 9.73 | 0.97 |
| Tao et al., 2017 ^19^ | 15.7℃ | 1100mm | 5750℃ | 4 | 100 | Green manure | 24.8 | 2.48 | 2.79 | 0.28 | 8.89 | 0.89 |
| Tian et al., 2017 ^20^ | 13.6℃ | 750 mm | 4571.9°C | 4 | 100 | ------ | 8.01 | 0.8 | 0.56 | 0.06 | 14.3 | 1.43 |
| Tian et al., 2017 ^20^ | 13.6℃ | 750 mm | 4571.9°C | 4 | 50 | ------ | 8.69 | 0.87 | 0.69 | 0.07 | 12.6 | 1.26 |
| Tian et al., 2017 ^20^ | 13.6℃ | 750 mm | 4571.9°C | 4 | 0 | ------ | 9.34 | 0.93 | 0.75 | 0.08 | 12.46 | 1.25 |
| Tian et al., 2017 ^20^ | 13.6℃ | 750 mm | 4571.9°C | 4 | 50 | ------ | 8.77 | 0.88 | 0.7 | 0.07 | 12.53 | 1.25 |
| Tian et al., 2017 ^20^ | 13.6℃ | 750 mm | 4571.9°C | 4 | 0 | ------ | 9.42 | 0.94 | 0.77 | 0.08 | 12.23 | 1.22 |
| Wang et al., 2010 ^21^ | 12℃ | 626.8mm | 3826℃ | 1 | 100 | ------ | 8.64 | 0.86 | 1 | 0.1 | 8.66 | 0.87 |
| Wang et al., 2010 ^21^ | 13℃ | 626.9mm | 3827℃ | 1 | 71 | ------ | 8.42 | 0.84 | 1.01 | 0.1 | 8.33 | 0.83 |
| Wang et al., 2010 ^21^ | 14℃ | 626.10mm | 3828℃ | 1 | 33 | ------ | 8.62 | 0.86 | 1.04 | 0.1 | 8.29 | 0.83 |
| Wang et al., 2010 ^21^ | 15℃ | 626.11mm | 3829℃ | 1 | 0 | ------ | 8.58 | 0.86 | 1.05 | 0.1 | 8.21 | 0.82 |
| Wang et al., 2012 ^22^ | 15.4℃ | 1054mm | 4933.7℃ | 4 | 100 | ------ | 11.08 | 1.11 | 2.14 | 0.21 | 5.18 | 0.52 |
| Wang et al., 2012 ^22^ | 15.4℃ | 1054mm | 4933.7℃ | 4 | 0 | ------ | 9.74 | 0.97 | 1.89 | 0.19 | 5.16 | 0.52 |
| Wang et al., 2012 ^22^ | 15.4℃ | 1054mm | 4933.7℃ | 4 | 100 | ------ | 8.41 | 0.84 | 1.84 | 0.18 | 4.57 | 0.46 |
| Wang et al., 2012 ^22^ | 15.4℃ | 1054mm | 4933.7℃ | 4 | 0 | ------ | 8.47 | 0.85 | 1.82 | 0.18 | 4.65 | 0.47 |
| Wang et al., 2015 ^23^ | 3.9℃ | 690.4 mm | 2700℃ | 1 | 100 | ------ | 5.83 | 0.58 | 0.28 | 0.03 | 20.87 | 2.09 |
| Wang et al., 2015 ^23^ | 3.9℃ | 690.4 mm | 2700℃ | 1 | 40 | ------ | 7.29 | 0.73 | 0.19 | 0.02 | 38.71 | 3.87 |
| Wang et al., 2015 ^23^ | 3.9℃ | 690.4 mm | 2700℃ | 1 | 20 | ------ | 3.81 | 0.38 | 0.19 | 0.02 | 20.25 | 2.02 |
| Wang et al., 2015 ^23^ | 3.9℃ | 690.4 mm | 2700℃ | 1 | 0 | ------ | 4.26 | 0.43 | 0.54 | 0.05 | 7.88 | 0.79 |
| Wang et al., 2019 ^24^ | 9.4 ℃ | 560mm | 2994℃ | 8 | 100 | -------- | 6.5 | 0.65 | 0.73 | 0.07 | 8.9 | 0.89 |
| Wang et al., 2019 ^24^ | 9.4 ℃ | 560mm | 2994℃ | 8 | 75 | -------- | 7 | 0.7 | 0.81 | 0.08 | 8.64 | 0.86 |
| Wang et al., 2019 ^24^ | 9.4 ℃ | 560mm | 2994℃ | 8 | 50 | -------- | 7.17 | 0.72 | 0.87 | 0.09 | 8.24 | 0.82 |
| Wang et al., 2019 ^24^ | 9.4 ℃ | 560mm | 2994℃ | 8 | 25 | -------- | 7.31 | 0.73 | 0.93 | 0.09 | 7.86 | 0.79 |
| Wang et al., 2019 ^24^ | 9.4 ℃ | 560mm | 2994℃ | 8 | 0 | -------- | 7.35 | 0.74 | 0.95 | 0.1 | 7.74 | 0.77 |
| Wang et al., 2019 ^25^ | 22.4℃ | 280mm | 2949.9℃ | 1 | 100 | ------ | 4.59 | 0.46 | 0.33 | 0.03 | 13.91 | 1.39 |
| Wang et al., 2019 ^25^ | 22.4℃ | 280mm | 2949.9℃ | 1 | 75 | ------ | 4.87 | 0.49 | 0.42 | 0.04 | 11.6 | 1.16 |
| Wang et al., 2019 ^25^ | 22.4℃ | 280mm | 2949.9℃ | 1 | 50 | ------ | 5.14 | 0.51 | 0.55 | 0.06 | 9.35 | 0.93 |
| Wang et al., 2019 ^25^ | 22.4℃ | 280mm | 2949.9℃ | 1 | 25 | ------ | 5.42 | 0.54 | 0.68 | 0.07 | 7.97 | 0.8 |
| Wang et al., 2019 ^25^ | 22.4℃ | 280mm | 2949.9℃ | 1 | 0 | ------ | 5.77 | 0.58 | 0.83 | 0.08 | 6.95 | 0.7 |
| Wang et al., 2019 ^25^ | 22.4℃ | 280mm | 2949.9℃ | 2 | 100 | ------ | 5.36 | 0.54 | 0.57 | 0.06 | 9.4 | 0.94 |
| Wang et al., 2019 ^25^ | 22.4℃ | 280mm | 2949.9℃ | 2 | 75 | ------ | 5.62 | 0.56 | 0.68 | 0.07 | 8.26 | 0.83 |
| Wang et al., 2019 ^25^ | 22.4℃ | 280mm | 2949.9℃ | 2 | 50 | ------ | 5.79 | 0.58 | 0.83 | 0.08 | 6.98 | 0.7 |
| Wang et al., 2019 ^25^ | 22.4℃ | 280mm | 2949.9℃ | 2 | 25 | ------ | 5.96 | 0.6 | 0.92 | 0.09 | 6.48 | 0.65 |
| Wang et al., 2019 ^25^ | 22.4℃ | 280mm | 2949.9℃ | 2 | 0 | ------ | 6.17 | 0.62 | 0.97 | 0.1 | 6.36 | 0.64 |
| Wang et al., 2019 ^25^ | 22.4℃ | 281mm | 2949.10℃ | 1 | 100 | Humic acid | 4.62 | 0.46 | 0.38 | 0.04 | 12.16 | 1.22 |
| Wang et al., 2019 ^25^ | 22.4℃ | 282mm | 2949.11℃ | 1 | 75 | ------ | 5 | 0.5 | 0.45 | 0.05 | 11.11 | 1.11 |
| Wang et al., 2019 ^25^ | 22.4℃ | 283mm | 2949.12℃ | 1 | 50 | ------ | 5.26 | 0.53 | 0.66 | 0.07 | 7.97 | 0.8 |
| Wang et al., 2019 ^25^ | 22.4℃ | 284mm | 2949.13℃ | 1 | 25 | ------ | 5.56 | 0.56 | 0.78 | 0.08 | 7.13 | 0.71 |
| Wang et al., 2019 ^25^ | 22.4℃ | 285mm | 2949.14℃ | 1 | 0 | ------ | 5.93 | 0.59 | 0.89 | 0.09 | 6.66 | 0.67 |
| Wang et al., 2019 ^25^ | 22.4℃ | 286mm | 2949.15℃ | 2 | 100 | Humic acid | 5.51 | 0.55 | 0.65 | 0.07 | 8.48 | 0.85 |
| Wang et al., 2019 ^25^ | 22.4℃ | 287mm | 2949.16℃ | 2 | 75 | ------ | 5.71 | 0.57 | 0.76 | 0.08 | 7.51 | 0.75 |
| Wang et al., 2019 ^25^ | 22.4℃ | 288mm | 2949.17℃ | 2 | 50 | ------ | 5.8 | 0.58 | 0.88 | 0.09 | 6.59 | 0.66 |
| Wang et al., 2019 ^25^ | 22.4℃ | 289mm | 2949.18℃ | 2 | 25 | ------ | 6.08 | 0.61 | 0.95 | 0.1 | 6.4 | 0.64 |
| Wang et al., 2019 ^25^ | 22.4℃ | 290mm | 2949.19℃ | 2 | 0 | ------ | 6.39 | 0.64 | 0.99 | 0.1 | 6.45 | 0.65 |
| Wang et al., 2020 ^26^ | 16.4℃ | 1363.5mm | 4820℃ | 1 | 100 | ------ | 18.27 | 1.83 | 0.78 | 0.08 | 23.42 | 2.34 |
| Wang et al., 2020 ^26^ | 16.4℃ | 1363.5mm | 4820℃ | 1 | 75 | ------ | 18.62 | 1.86 | 0.81 | 0.08 | 22.99 | 2.3 |
| Wang et al., 2020 ^26^ | 16.4℃ | 1363.5mm | 4820℃ | 1 | 50 | ------ | 19.66 | 1.97 | 0.84 | 0.08 | 23.41 | 2.34 |
| Wang et al., 2020 ^26^ | 16.4℃ | 1363.5mm | 4820℃ | 1 | 0 | ------ | 19.6 | 1.96 | 0.88 | 0.09 | 22.28 | 2.23 |
| Wang et al., 2020 ^26^ | 16.4℃ | 1363.5mm | 4820℃ | 1 | 100 | ------ | 17.86 | 1.79 | 0.71 | 0.07 | 25.16 | 2.52 |
| Wang et al., 2020 ^26^ | 16.4℃ | 1363.5mm | 4820℃ | 1 | 75 | ------ | 17.81 | 1.78 | 0.74 | 0.07 | 24.06 | 2.41 |
| Wang et al., 2020 ^26^ | 16.4℃ | 1363.5mm | 4820℃ | 1 | 50 | ------ | 19.26 | 1.93 | 0.77 | 0.08 | 25.01 | 2.5 |
| Wang et al., 2020 ^26^ | 16.4℃ | 1363.5mm | 4820℃ | 1 | 0 | ------ | 17.98 | 1.8 | 0.82 | 0.08 | 21.93 | 2.19 |
| Wang et al., 2020 ^26^ | 16.4℃ | 1363.5mm | 4820℃ | 1 | 100 | biochar | 23.95 | 2.4 | 0.78 | 0.08 | 30.71 | 3.07 |
| Wang et al., 2020 ^26^ | 16.4℃ | 1363.5mm | 4820℃ | 1 | 75 | ------ | 23.49 | 2.35 | 0.82 | 0.08 | 28.65 | 2.86 |
| Wang et al., 2020 ^26^ | 16.4℃ | 1363.5mm | 4820℃ | 1 | 50 | ------ | 23.84 | 2.38 | 0.84 | 0.08 | 28.38 | 2.84 |
| Wang et al., 2020 ^26^ | 16.4℃ | 1363.5mm | 4820℃ | 1 | 0 | ------ | 25 | 2.5 | 0.89 | 0.09 | 28.09 | 2.81 |
| Wang et al., 2020 ^26^ | 16.4℃ | 1363.5mm | 4820℃ | 1 | 100 | biochar | 19.43 | 1.94 | 0.71 | 0.07 | 27.37 | 2.74 |
| Wang et al., 2020 ^26^ | 16.4℃ | 1363.5mm | 4820℃ | 1 | 75 | ------ | 19.84 | 1.98 | 0.75 | 0.08 | 26.45 | 2.64 |
| Wang et al., 2020 ^26^ | 16.4℃ | 1363.5mm | 4820℃ | 1 | 50 | ------ | 20.07 | 2.01 | 0.78 | 0.08 | 25.73 | 2.57 |
| Wang et al., 2020 ^26^ | 16.4℃ | 1363.5mm | 4820℃ | 1 | 0 | ------ | 19.72 | 1.97 | 0.82 | 0.08 | 24.05 | 2.4 |
| Xie et al., 2015 ^27^ | 17.2℃ | 1680mm | 5644℃ | 1 | 0 | - | 22.5 | 4.9 | 2.04 | 0.44 | 11.04 | 1.1 |
| Xie et al., 2015 ^27^ | 17.2℃ | 1680mm | 5644℃ | 1 | 20 | Commercial organic fertilizer | 17.47 | 3.57 | 2.18 | 0.44 | 8 | 0.8 |
| Xie et al., 2015 ^27^ | 17.2℃ | 1680mm | 5644℃ | 1 | 20 | Commercial organic fertilizer | 18.22 | 8.02 | 2.16 | 0.13 | 8.45 | 0.84 |
| Xie et al., 2015 ^27^ | 17.2℃ | 1680mm | 5644℃ | 1 | 20 | Commercial organic fertilizer | 18.37 | 7.13 | 2.12 | 0.31 | 8.66 | 0.87 |
| Xie et al., 2015 ^27^ | 17.2℃ | 1680mm | 5644℃ | 1 | 0 | - | 18.82 | 0.89 | 1.95 | 0.31 | 9.65 | 0.97 |
| Xie et al., 2015 ^27^ | 17.2℃ | 1680mm | 5644℃ | 1 | 20 | Commercial organic fertilizer | 17.79 | 1.78 | 2.01 | 0.19 | 8.83 | 0.88 |
| Xie et al., 2015 ^27^ | 17.2℃ | 1680mm | 5644℃ | 1 | 20 | Commercial organic fertilizer | 18.39 | 1.34 | 2.05 | 0.28 | 8.97 | 0.9 |
| Xie et al., 2015 ^27^ | 17.2℃ | 1680mm | 5644℃ | 1 | 20 | Commercial organic fertilizer | 18.09 | 1.34 | 1.97 | 0.28 | 9.18 | 0.92 |
| Yang et al., 2019 ^28^ | 0.1℃ | 570mm | 2571.2℃ | 1 | 100 | - | 21.92 | 0.72 | 1.8 | 0.35 | 12.18 | 1.22 |
| Yang et al., 2019 ^28^ | 0.1℃ | 570mm | 2571.2℃ | 1 | 0 | - | 23.9 | 6.01 | 2.11 | 0.19 | 11.33 | 1.13 |
| Yang et al., 2019 ^28^ | 0.1℃ | 570mm | 2571.2℃ | 1 | 0 | cow dung | 23.56 | 2.38 | 2.31 | 0.24 | 10.2 | 1.02 |
| Yang et al., 2019 ^28^ | 0.1℃ | 570mm | 2571.2℃ | 1 | 0 | cow dung | 23.88 | 2.9 | 2.62 | 0.09 | 9.11 | 0.91 |
| Yang et al., 2019 ^28^ | 0.1℃ | 570mm | 2571.2℃ | 1 | 30 | cow dung | 22.22 | 0.93 | 2.18 | 0.16 | 10.19 | 1.02 |
| Yang et al., 2019 ^28^ | 0.1℃ | 570mm | 2571.2℃ | 1 | 50 | cow dung | 22.57 | 3.52 | 2.52 | 0.17 | 8.96 | 0.9 |
| Yang et al., 2019 ^28^ | 0.1℃ | 570mm | 2571.2℃ | 1 | 100 | - | 21.71 | 2.8 | 1.79 | 0.21 | 12.13 | 1.21 |
| Yang et al., 2019 ^28^ | 0.1℃ | 570mm | 2571.2℃ | 1 | 0 | - | 22.93 | 0.41 | 2.07 | 0.23 | 11.08 | 1.11 |
| Yang et al., 2019 ^28^ | 0.1℃ | 570mm | 2571.2℃ | 1 | 0 | cow dung | 21.69 | 1.55 | 2.14 | 0.16 | 10.13 | 1.01 |
| Yang et al., 2019 ^28^ | 0.1℃ | 570mm | 2571.2℃ | 1 | 0 | cow dung | 22.7 | 3.52 | 2.26 | 0.17 | 10.04 | 1 |
| Yang et al., 2019 ^28^ | 0.1℃ | 570mm | 2571.2℃ | 1 | 30 | cow dung | 22.88 | 4.14 | 2.36 | 0.14 | 9.69 | 0.97 |
| Yang et al., 2019 ^28^ | 0.1℃ | 570mm | 2571.2℃ | 1 | 50 | cow dung | 22.57 | 3 | 2.21 | 0.09 | 10.21 | 1.02 |
| Yu et al., 2015 ^29^ | 11.4℃ | 577mm | 4312℃ | 3 | 100 | ------ | 8.12 | 0.81 | 0.81 | 0.08 | 10.02 | 1 |
| Yu et al., 2015 ^29^ | 11.4℃ | 577mm | 4312℃ | 3 | 75 | ------ | 8.41 | 0.84 | 0.84 | 0.08 | 10.01 | 1 |
| Yu et al., 2015 ^29^ | 11.4℃ | 577mm | 4312℃ | 3 | 50 | ------ | 9.11 | 0.91 | 0.87 | 0.09 | 10.47 | 1.05 |
| Yu et al., 2015 ^29^ | 11.4℃ | 577mm | 4312℃ | 3 | 25 | ------ | 8.53 | 0.85 | 0.84 | 0.08 | 10.15 | 1.02 |
| Yu et al., 2015 ^29^ | 11.4℃ | 577mm | 4312℃ | 3 | 0 | ------ | 8.06 | 0.81 | 0.88 | 0.09 | 9.16 | 0.92 |
| Yu et al., 2015 ^29^ | 11.4℃ | 577mm | 4312℃ | 3 | 100 | Pig manure | 8.47 | 0.85 | 0.89 | 0.09 | 9.51 | 0.95 |
| Yu et al., 2015 ^29^ | 11.4℃ | 577mm | 4312℃ | 3 | 75 | ------ | 9.8 | 0.98 | 0.95 | 0.1 | 10.32 | 1.03 |
| Yu et al., 2015 ^29^ | 11.4℃ | 577mm | 4312℃ | 3 | 50 | ------ | 10.03 | 1 | 0.96 | 0.1 | 10.45 | 1.05 |
| Yu et al., 2015 ^29^ | 11.4℃ | 577mm | 4312℃ | 3 | 25 | ------ | 9.86 | 0.99 | 0.99 | 0.1 | 9.96 | 1 |
| Yu et al., 2015 ^29^ | 11.4℃ | 577mm | 4312℃ | 3 | 0 | ------ | 9.74 | 0.97 | 1.06 | 0.11 | 9.19 | 0.92 |
| Yu et al., 2015 ^29^ | 11.5℃ | 577 mm | 4448℃ | 4 | 100 | ------ | 8.12 | 0.81 | 0.81 | 0.08 | 10.02 | 1 |
| Yu et al., 2015 ^29^ | 11.5℃ | 577 mm | 4448℃ | 4 | 75 | ------ | 8.41 | 0.84 | 0.84 | 0.08 | 10.01 | 1 |
| Yu et al., 2015 ^29^ | 11.5℃ | 577 mm | 4448℃ | 4 | 50 | ------ | 9.11 | 0.91 | 0.87 | 0.09 | 10.47 | 1.05 |
| Yu et al., 2015 ^29^ | 11.5℃ | 577 mm | 4448℃ | 4 | 25 | ------ | 8.53 | 0.85 | 0.84 | 0.08 | 10.15 | 1.02 |
| Yu et al., 2015 ^29^ | 11.5℃ | 577 mm | 4448℃ | 4 | 0 | ------ | 8.06 | 0.81 | 0.88 | 0.09 | 9.16 | 0.92 |
| Yu et al., 2015 ^29^ | 11.5℃ | 577 mm | 4448℃ | 4 | 100 | Pig manure | 8.47 | 0.85 | 0.89 | 0.09 | 9.51 | 0.95 |
| Yu et al., 2015 ^29^ | 11.5℃ | 577 mm | 4448℃ | 4 | 75 | ------ | 9.8 | 0.98 | 0.95 | 0.1 | 10.32 | 1.03 |
| Yu et al., 2015 ^29^ | 11.5℃ | 577 mm | 4448℃ | 4 | 50 | ------ | 10.03 | 1 | 0.96 | 0.1 | 10.45 | 1.05 |
| Yu et al., 2015 ^29^ | 11.5℃ | 577 mm | 4448℃ | 4 | 25 | ------ | 9.86 | 0.99 | 0.99 | 0.1 | 9.96 | 1 |
| Yu et al., 2015 ^29^ | 11.5℃ | 577 mm | 4448℃ | 4 | 0 | ------ | 9.74 | 0.97 | 1.06 | 0.11 | 9.19 | 0.92 |
| Yu et al., 2019 ^30^ | 5.8℃ | 474 mm | 3046.8℃ | 6 | 100 | ------ | 17.5 | 3.15 | 1.43 | 0.05 | 12.24 | 1.22 |
| Yu et al., 2019 ^30^ | 5.8℃ | 474 mm | 3046.8℃ | 6 | 50 | ------ | 16.8 | 0.48 | 1.48 | 0.03 | 11.35 | 1.14 |
| Yu et al., 2019 ^30^ | 5.8℃ | 474 mm | 3046.8℃ | 6 | 0 | ------ | 13.6 | 0.78 | 1.46 | 0.09 | 9.32 | 0.93 |
| Yu et al., 2019 ^30^ | 5.8℃ | 474 mm | 3046.8℃ | 6 | 100 | ------ | 12.9 | 1.56 | 1.18 | 0.05 | 10.93 | 1.09 |
| Yu et al., 2019 ^30^ | 5.8℃ | 474 mm | 3046.8℃ | 6 | 50 | ------ | 10.7 | 1.13 | 1.14 | 0.05 | 9.39 | 0.94 |
| Yu et al., 2019 ^30^ | 5.8℃ | 474 mm | 3046.8℃ | 6 | 0 | ------ | 10.8 | 0.64 | 1.1 | 0.1 | 9.82 | 0.98 |
| Yu et al., 2019 ^30^ | 5.8℃ | 474 mm | 3046.8℃ | 6 | 100 | ------ | 7.16 | 1.45 | 1.13 | 0.05 | 6.34 | 0.63 |
| Yu et al., 2019 ^30^ | 5.8℃ | 474 mm | 3046.8℃ | 6 | 50 | ------ | 7.83 | 0.57 | 1.09 | 0.02 | 7.18 | 0.72 |
| Yu et al., 2019 ^30^ | 5.8℃ | 474 mm | 3046.8℃ | 6 | 0 | ------ | 9.08 | 1.63 | 1.03 | 0.17 | 8.82 | 0.88 |
| Yuan et al., 2017 ^31^ | 15℃ | 700mm | 4500℃ | 3 | 100 | ------ | 5.06 | 1.73 | 1.4 | 0.14 | 3.6 | 0.36 |
| Yuan et al., 2017 ^31^ | 15℃ | 700mm | 4500℃ | 3 | 0 | ------ | 6.79 | 1.8 | 1.57 | 0.17 | 4.33 | 0.43 |
| Yuan et al., 2017 ^31^ | 15℃ | 700mm | 4500℃ | 3 | 100 | Humic acid | 7.96 | 1.73 | 1.49 | 0.14 | 5.35 | 0.54 |
| Yuan et al., 2017 ^31^ | 15℃ | 700mm | 4500℃ | 3 | 0 | Humic acid | 8.24 | 1.73 | 1.59 | 0.16 | 5.2 | 0.52 |
| Yuan et al., 2017 ^31^ | 15℃ | 700mm | 4500℃ | 3 | 15 | Humic acid | 8.83 | 1.8 | 1.66 | 0.17 | 5.33 | 0.53 |
| Yuan et al., 2017 ^31^ | 15℃ | 700mm | 4500℃ | 3 | 30 | Humic acid | 8.04 | 1.8 | 1.62 | 0.16 | 4.95 | 0.5 |
| Yuan et al., 2017 ^31^ | 15℃ | 805.8mm | 5296.7℃ | 3 | 100 | ------ | 3.4 | 1.02 | 0.95 | 0.08 | 3.56 | 0.36 |
| Yuan et al., 2017 ^31^ | 15℃ | 805.8mm | 5296.7℃ | 3 | 0 | ------ | 4.76 | 0.96 | 1.08 | 0.11 | 4.41 | 0.44 |
| Yuan et al., 2017 ^31^ | 15℃ | 805.8mm | 5296.7℃ | 3 | 100 | Humic acid | 5.56 | 0.91 | 1.18 | 0.13 | 4.73 | 0.47 |
| Yuan et al., 2017 ^31^ | 15℃ | 805.8mm | 5296.7℃ | 3 | 0 | Humic acid | 5.97 | 1.02 | 1.23 | 0.11 | 4.87 | 0.49 |
| Yuan et al., 2017 ^31^ | 15℃ | 805.8mm | 5296.7℃ | 3 | 15 | Humic acid | 6.34 | 0.96 | 1.31 | 0.14 | 4.83 | 0.48 |
| Yuan et al., 2017 ^31^ | 15℃ | 805.8mm | 5296.7℃ | 3 | 30 | Humic acid | 6.02 | 0.96 | 1.26 | 0.12 | 4.79 | 0.48 |
| Yuan et al., 2017 ^32^ | 13.4℃ | 600 mm | 3500℃ | 2 | 100 | ------ | 10.82 | 1.08 | 0.71 | 0.07 | 15.24 | 1.52 |
| Yuan et al., 2017 ^32^ | 13.4℃ | 600 mm | 3500℃ | 2 | 50 | ------ | 11.33 | 1.13 | 0.8 | 0.08 | 14.16 | 1.42 |
| Yuan et al., 2017 ^32^ | 13.4℃ | 600 mm | 3500℃ | 2 | 25 | ------ | 11.36 | 1.14 | 0.77 | 0.08 | 14.75 | 1.48 |
| Yuan et al., 2017 ^32^ | 13.4℃ | 600 mm | 3500℃ | 2 | 0 | ------ | 12.83 | 1.28 | 0.84 | 0.08 | 15.27 | 1.53 |
| Yuan et al., 2017 ^32^ | 13.4℃ | 600 mm | 3500℃ | 2 | 50 | biochar | 12.43 | 1.24 | 0.85 | 0.09 | 14.62 | 1.46 |
| Yuan et al., 2017 ^32^ | 13.4℃ | 600 mm | 3500℃ | 2 | 25 | ------ | 12.17 | 1.22 | 0.92 | 0.09 | 13.23 | 1.32 |
| Yuan et al., 2017 ^32^ | 13.4℃ | 600 mm | 3500℃ | 2 | 0 | ------ | 12.08 | 1.21 | 0.86 | 0.09 | 14.05 | 1.4 |
| Yuan et al., 2017 ^32^ | 13.4℃ | 600 mm | 3500℃ | 2 | 50 | biochar | 14.33 | 1.43 | 1 | 0.1 | 14.33 | 1.43 |
| Yuan et al., 2017 ^32^ | 13.4℃ | 600 mm | 3500℃ | 2 | 25 | ------ | 13.67 | 1.37 | 1.12 | 0.11 | 12.21 | 1.22 |
| Yuan et al., 2017 ^32^ | 13.4℃ | 600 mm | 3500℃ | 2 | 0 | ------ | 12.67 | 1.27 | 0.97 | 0.1 | 13.06 | 1.31 |
| Yuan et al., 2017 ^32^ | 13.4℃ | 600 mm | 3500℃ | 2 | 50 | biochar | 14.06 | 1.41 | 1.21 | 0.12 | 11.62 | 1.16 |
| Yuan et al., 2017 ^32^ | 13.4℃ | 600 mm | 3500℃ | 2 | 25 | ------ | 14.08 | 1.41 | 1.21 | 0.12 | 11.64 | 1.16 |
| Yuan et al., 2017 ^32^ | 13.4℃ | 600 mm | 3500℃ | 2 | 0 | ------ | 13.49 | 1.35 | 1.28 | 0.13 | 10.54 | 1.05 |
| Zhang et al., 2014 ^33^ | 13.8 ℃ | 673mm | 4605.5℃ | 7 | 33 | ----- | 10.13 | 1.01 | 0.98 | 0.1 | 10.35 | 1.03 |
| Zhang et al., 2014 ^33^ | 13.8 ℃ | 673mm | 4605.5℃ | 7 | 16 | ----- | 11.01 | 1.1 | 1.02 | 0.1 | 10.77 | 1.08 |
| Zhang et al., 2014 ^33^ | 13.8 ℃ | 673mm | 4605.5℃ | 7 | 0 | ------ | 10.76 | 1.08 | 1.02 | 0.1 | 10.6 | 1.06 |
| Zhang et al., 2014 ^33^ | 13.8 ℃ | 673mm | 4605.5℃ | 7 | 33 | manure | 11.13 | 1.11 | 0.91 | 0.09 | 12.3 | 1.23 |
| Zhang et al., 2014 ^33^ | 13.8 ℃ | 673mm | 4605.5℃ | 7 | 16 | ------ | 10.6 | 1.06 | 0.95 | 0.1 | 11.15 | 1.11 |
| Zhang et al., 2014 ^33^ | 13.8 ℃ | 673mm | 4605.5℃ | 7 | 0 | ------ | 10.57 | 1.06 | 1.01 | 0.1 | 10.51 | 1.05 |
| Zhang et al., 2014 ^33^ | 13.8 ℃ | 673mm | 4605.5℃ | 7 | 33 | manure | 11.13 | 1.11 | 0.95 | 0.09 | 11.74 | 1.17 |
| Zhang et al., 2014 ^33^ | 13.8 ℃ | 673mm | 4605.5℃ | 7 | 16 | ------ | 10 | 1 | 1.02 | 0.1 | 9.81 | 0.98 |
| Zhang et al., 2014 ^33^ | 13.8 ℃ | 673mm | 4605.5℃ | 7 | 0 | ------- | 10.38 | 1.04 | 0.98 | 0.1 | 10.58 | 1.06 |
| Zhang et al., 2014 ^33^ | 13.8 ℃ | 673mm | 4605.5℃ | 7 | 33 | manure | 10.98 | 1.1 | 1.05 | 0.11 | 10.45 | 1.04 |
| Zhang et al., 2014 ^33^ | 13.8 ℃ | 673mm | 4605.5℃ | 7 | 16 | - | 11.32 | 1.13 | 1.02 | 0.1 | 11.11 | 1.11 |
| Zhang et al., 2014 ^33^ | 13.8 ℃ | 673mm | 4605.5℃ | 7 | 0 | - | 11.45 | 1.15 | 1.09 | 0.11 | 10.49 | 1.05 |
| Zhang et al., 2014 ^33^ | 13.8 ℃ | 673mm | 4605.5℃ | 7 | 33 | manure | 12.2 | 1.22 | 1.21 | 0.12 | 10.11 | 1.01 |
| Zhang et al., 2014 ^33^ | 13.8 ℃ | 673mm | 4605.5℃ | 7 | 16 | -------- | 11.49 | 1.15 | 1.08 | 0.11 | 10.69 | 1.07 |
| Zhang et al., 2014 ^33^ | 13.8 ℃ | 673mm | 4605.5℃ | 7 | 0 | -------- | 12.51 | 1.25 | 1.13 | 0.11 | 11.1 | 1.11 |
| Zhang et al., 2014 ^33^ | 15℃ | 960mm | 4776℃ | 4 | 100 | ------ | 4.76 | 0.48 | 0.85 | 0.09 | 5.6 | 0.56 |
| Zhang et al., 2014 ^33^ | 15℃ | 960mm | 4776℃ | 4 | 0 | ------ | 5.22 | 0.52 | 1.07 | 0.11 | 4.88 | 0.49 |
| Zhang et al., 2014 ^33^ | 15℃ | 960mm | 4776℃ | 4 | 50 | ------ | 5.2 | 0.52 | 0.81 | 0.08 | 6.42 | 0.64 |
| Zhang et al., 2014 ^33^ | 15℃ | 960mm | 4776℃ | 4 | 0 | ------ | 5.16 | 0.52 | 1.03 | 0.1 | 5.01 | 0.5 |
| Zhang et al., 2014 ^33^ | 15℃ | 960mm | 4776℃ | 4 | 50 | ------ | 5.26 | 0.53 | 0.78 | 0.08 | 6.74 | 0.67 |
| Zhang et al., 2014 ^33^ | 15.1℃ | 497.5 mm | 3553℃ | 4 | 0 | ------ | 6.72 | 0.67 | 1.25 | 0.13 | 5.38 | 0.54 |
| Zhang et al., 2014 ^33^ | 15.1℃ | 497.5 mm | 3553℃ | 4 | 30 | ------ | 6.79 | 0.68 | 1.25 | 0.13 | 5.43 | 0.54 |
| Zhang et al., 2014 ^33^ | 15.1℃ | 497.5 mm | 3553℃ | 4 | 0 | ------ | 6.8 | 0.68 | 1.13 | 0.11 | 6.02 | 0.6 |
| Zhang et al., 2014 ^33^ | 15.1℃ | 497.5 mm | 3553℃ | 4 | 30 | ------ | 6.54 | 0.65 | 1.26 | 0.13 | 5.19 | 0.52 |
| Zhang et al., 2014 ^33^ | 15.1℃ | 497.5 mm | 3553℃ | 4 | 100 | ------ | 5.9 | 0.59 | 1.24 | 0.12 | 4.76 | 0.48 |
| Zhang et al., 2014 ^33^ | 16．5℃ | 1041.8 mm | 5300℃ | 7 | 100 | ------ | 8.44 | 0.84 | 1.06 | 0.11 | 7.97 | 0.8 |
| Zhang et al., 2014 ^33^ | 16．5℃ | 1041.8 mm | 5300℃ | 7 | 0 | ------ | 9.28 | 0.93 | 1.14 | 0.11 | 8.17 | 0.82 |
| Zhang et al., 2014 ^33^ | 16．5℃ | 1041.8 mm | 5300℃ | 7 | 30 | ------ | 8.12 | 0.81 | 1.19 | 0.12 | 6.84 | 0.68 |
| Zhang et al., 2014 ^33^ | 16．5℃ | 1041.8 mm | 5300℃ | 7 | 50 | ------ | 8.12 | 0.81 | 1.24 | 0.12 | 6.55 | 0.66 |
| Zhang et al., 2014 ^33^ | 16．5℃ | 1041.8 mm | 5300℃ | 7 | 0 | ------ | 9.28 | 0.93 | 1.29 | 0.13 | 7.19 | 0.72 |
| Zhang et al., 2014 ^33^ | 16．5℃ | 1041.8 mm | 5300℃ | 7 | 30 | ------ | 8.21 | 0.82 | 1.35 | 0.14 | 6.08 | 0.61 |
| Zhang et al., 2014 ^33^ | 16．5℃ | 1041.8 mm | 5300℃ | 7 | 50 | ------ | 7.89 | 0.79 | 1.41 | 0.14 | 5.59 | 0.56 |
| Zhang et al., 2014 ^33^ | 12．5℃ | 582.8 mm | 4500℃ | 4 | 100 | ------ | 13.94 | 1.39 | 1.13 | 0.11 | 12.33 | 1.23 |
| Zhang et al., 2014 ^33^ | 12．5℃ | 582.8 mm | 4500℃ | 4 | 0 | ------ | 13.35 | 1.33 | 1.26 | 0.13 | 10.59 | 1.06 |
| Zhang et al., 2014 ^33^ | 12．5℃ | 582.8 mm | 4500℃ | 4 | 20 | ------ | 13.25 | 1.33 | 1.3 | 0.13 | 10.19 | 1.02 |
| Zhang et al., 2014 ^33^ | 12．5℃ | 582.8 mm | 4500℃ | 4 | 40 | ------ | 14.41 | 1.44 | 1.25 | 0.13 | 11.53 | 1.15 |
| Zhang et al., 2015 ^34^ | 15.6℃ | 1085mm | 5190℃ | 1 | 100 | ------ | 20.04 | 0.47 | 1.3 | 0.02 | 15.41 | 1.54 |
| Zhang et al., 2015 ^34^ | 15.6℃ | 1085mm | 5190℃ | 1 | 0 | ------ | 19.89 | 0.8 | 1.78 | 0.16 | 11.18 | 1.12 |
| Zhang et al., 2015 ^34^ | 15.6℃ | 1085mm | 5190℃ | 1 | 20 | ------ | 19.89 | 0.7 | 1.89 | 0.07 | 10.53 | 1.05 |
| Zhang et al., 2015 ^34^ | 15.6℃ | 1085mm | 5190℃ | 1 | 20 | Organic fertilizer | 21.36 | 0.55 | 1.88 | 0.28 | 11.36 | 1.14 |
| Zhang et al., 2015 ^34^ | 15.6℃ | 1085mm | 5190℃ | 1 | 40 | Organic fertilizer | 21.03 | 0.44 | 1.76 | 0.24 | 11.95 | 1.19 |
| Zhang et al., 2015 ^34^ | 15.6℃ | 1085mm | 5190℃ | 1 | 100 | ------ | 18.88 | 0.51 | 1.17 | 0.07 | 16.14 | 1.61 |
| Zhang et al., 2015 ^34^ | 15.6℃ | 1085mm | 5190℃ | 1 | 0 | ------ | 20.35 | 0.51 | 1.48 | 0.26 | 13.75 | 1.38 |
| Zhang et al., 2015 ^34^ | 15.6℃ | 1085mm | 5190℃ | 1 | 20 | ------ | 19.62 | 0.42 | 1.59 | 0.07 | 12.34 | 1.23 |
| Zhang et al., 2015 ^34^ | 15.6℃ | 1085mm | 5190℃ | 1 | 20 | Organic fertilizer | 20.58 | 0.42 | 1.91 | 0.12 | 10.78 | 1.08 |
| Zhang et al., 2015 ^34^ | 15.6℃ | 1085mm | 5190℃ | 1 | 40 | Organic fertilizer | 18.93 | 0.58 | 1.89 | 0.1 | 10.01 | 1 |
| Zhang et al., 2017 ^35^ | 17.2℃ | 1680mm | 5644℃ | 2 | 0 | - | 18.06 | 3.08 | 1.91 | 0.11 | 9.45 | 0.94 |
| Zhang et al., 2017 ^35^ | 17.2℃ | 1680mm | 5644℃ | 2 | 20 | Commercial organic fertilizer | 18.06 | 4.93 | 2.01 | 0.15 | 9 | 0.9 |
| Zhang et al., 2017 ^35^ | 17.2℃ | 1680mm | 5644℃ | 2 | 20 | Commercial organic fertilizer | 18.37 | 2.47 | 1.99 | 0.33 | 9.25 | 0.93 |
| Zhang et al., 2017 ^35^ | 17.2℃ | 1680mm | 5644℃ | 2 | 20 | Commercial organic fertilizer | 18.37 | 3.08 | 1.92 | 0.18 | 9.56 | 0.96 |
| Zhang et al., 2017 ^35^ | 17.2℃ | 1680mm | 5644℃ | 2 | 0 | - | 18.17 | 3.7 | 1.92 | 0.11 | 9.45 | 0.95 |
| Zhang et al., 2017 ^35^ | 17.2℃ | 1680mm | 5644℃ | 2 | 20 | Commercial organic fertilizer | 17.55 | 3.7 | 1.93 | 0.18 | 9.08 | 0.91 |
| Zhang et al., 2017 ^35^ | 17.2℃ | 1680mm | 5644℃ | 2 | 20 | Commercial organic fertilizer | 18.27 | 3.08 | 1.94 | 0.11 | 9.4 | 0.94 |
| Zhang et al., 2017 ^35^ | 17.2℃ | 1680mm | 5644℃ | 2 | 20 | Commercial organic fertilizer | 19.1 | 3.08 | 1.93 | 0.18 | 9.88 | 0.99 |
| Zhang et al., 2017 ^35^ | 17.2℃ | 1680mm | 5644℃ | 3 | 0 | - | 18.79 | 1.85 | 1.94 | 0.07 | 9.67 | 0.97 |
| Zhang et al., 2017 ^35^ | 17.2℃ | 1680mm | 5644℃ | 3 | 20 | Commercial organic fertilizer | 19.72 | 3.08 | 2.12 | 0.11 | 9.29 | 0.93 |
| Zhang et al., 2017 ^35^ | 17.2℃ | 1680mm | 5644℃ | 3 | 20 | Commercial organic fertilizer | 21.16 | 1.85 | 1.99 | 0.11 | 10.66 | 1.07 |
| Zhang et al., 2017 ^35^ | 17.2℃ | 1680mm | 5644℃ | 3 | 20 | Commercial organic fertilizer | 20.95 | 2.47 | 2.06 | 0.18 | 10.18 | 1.02 |
| Zhang et al., 2017 ^35^ | 17.2℃ | 1680mm | 5644℃ | 3 | 0 | - | 20.75 | 3.08 | 2.12 | 0.04 | 9.78 | 0.98 |
| Zhang et al., 2017 ^35^ | 17.2℃ | 1680mm | 5644℃ | 3 | 20 | Commercial organic fertilizer | 20.95 | 3.08 | 2.15 | 0.18 | 9.73 | 0.97 |
| Zhang et al., 2017 ^35^ | 17.2℃ | 1680mm | 5644℃ | 3 | 20 | Commercial organic fertilizer | 21.47 | 1.85 | 2.23 | 0.11 | 9.64 | 0.96 |
| Zhang et al., 2017 ^35^ | 17.2℃ | 1680mm | 5644℃ | 3 | 20 | Commercial organic fertilizer | 22.3 | 3.7 | 2.29 | 0.18 | 9.74 | 0.97 |
| Zuo et al., 2012 ^36^ | 17.8℃ | 1662 mm | 5432.2℃ | 2 | 100 | ------ | 15.3 | 0.23 | 1.49 | 0.02 | 10.28 | 1.03 |
| Zuo et al., 2012 ^36^ | 17.9℃ | 1663 mm | 5432.3℃ | 2 | 100 | straw | 15.98 | 0.35 | 1.71 | 0.22 | 9.32 | 0.93 |
| Zuo et al., 2012 ^36^ | 17.10℃ | 1664 mm | 5432.4℃ | 2 | 0 | straw | 16.66 | 0.23 | 1.76 | 0.28 | 9.44 | 0.94 |
| Zuo et al., 2012 ^36^ | 17.11℃ | 1665 mm | 5432.5℃ | 2 | 18 | straw | 15.83 | 0.35 | 1.71 | 0.3 | 9.24 | 0.92 |
| Zuo et al., 2012 ^36^ | 17.12℃ | 1666 mm | 5432.6℃ | 2 | 18 | straw | 16.51 | 0.23 | 1.65 | 0.08 | 10.02 | 1 |
| Zuo et al., 2012 ^36^ | 17.13℃ | 1667 mm | 5432.7℃ | 2 | 18 | straw | 15.34 | 0.81 | 1.58 | 0.13 | 9.7 | 0.97 |
| Zuo et al., 2012 ^36^ | 17.14℃ | 1668 mm | 5432.8℃ | 2 | 18 | straw | 16.36 | 0.23 | 1.67 | 0.2 | 9.78 | 0.98 |

Reference

1. Bei S K, Zhang Y L, Li T T, Christie P, Li X L, Zhang J L, 2018. Response of the soil microbial community to different fertilizer inputs in a wheat-maize rotation on a calcareous soil. Agriculture, Ecosystems & Environment, 260: 58-69.
2. Chen J, 2017. The regulation of tillage practice and nitrogen rate for improving soil quality and grain yield of winter wheat. Shandong Agricultural University.
3. Chen L, Li F, Li W, Ning Q, Li J W, Zhang J B, Ma D H, Zhang C Z, 2020. Organic amendment mitigates the negative impacts of mineral fertilization. Applied Soil Ecology, 150: 103457.
4. Cheng B Y, Han Y N, Li Y Y, Jiang C J, 2014. Effects of different fertilization patterns on soil nutrient, tea quality and yield components. Chinese Journal of Eco-Agriculture, 22(5): 525-533.
5. He L, Li Z, Zhe K, Yang H M, 2017. Effects on soil quality of biochar and straw amendment in conjunction with chemical fertilizers - effects on soil quality of biochar and straw amendment in conjunction with chemical fertilizers. Journal of Integrative Agriculture, 16(3): 704-712.
6. Hou H Q, Ji J H, Liu G R, Liu Y R, Liu X M, Cheng Z X, Yang J C, Wen S L, 2012. On the mode of nitrogen-reduction in double-rice cropping region in red soil area of south area. Chinese journal of rice science, 26(5): 555-562.
7. Lan M J, 2017. Response of dry farmland soil organic carbon and its liable organic carbon fractions to nitrogen fertilization in Loess Plateau of central Gansu province.
8. Li J, Peng J L, Kang J, Zhou L J, Lin W F, 2012. Effects of nitrogen-reduction on the soil profile nutrient distribution in paddy fields. Chinese Journal of Tropical Crops, 33(8): 1378-1383.
9. Li R, Tao R, Ling N, Chu G X, 2017. Chemical, organic and bio-fertilizer management practices effect on soil physicochemical property and antagonistic bacteria abundance of a cotton field: implications for soil biological quality. Soil & Tillage Research, 167: 30-38.
10. Liang L, 2019. Effects of combined application of organic and inorganic fertilizers on soil nutrient availability and enzyme activity in rainfed wheat field. Northwest A & F University.
11. Liu Z X, 2013. A preliminary study on combined application of biochar and nitrogen fertility of typical upland red soil. Nanjing Agricultural University.
12. Liu Y B, Zhu Q F, Bi B J, Wang R F, 2016. Effect of N, P and K fertilizers on growth of clover nitrogen-fixing rhizobia and soil fertility after plantation, Agricultural Science and Technology, 17(4): 906-911.
13. Liu Z, Sun K, Liu W T, Gao T P, Li G, Han H F, Li Z J, Ning T Y, 2020. Responses of soil carbon, nitrogen, and wheat and maize productivity to 10 years of decreased nitrogen fertilizer under contrasting tillage systems. Soil & Tillage Research,196: 104444.
14. Lu B L, Bao X G, Zhang J D, Yang X Q, Yang W Y, Li Q F, Cao W D, 2015. Effects of intercropping green manure forages and nitrogen-reduction on corn yield and soil fertility in Hexi Oasis Irrigation. Agricultural research in the Arid Areas, 33(2): 170-175.
15. Ma S T, 2015. Effects of straw returning and reducing nitrogen fertilizer on carbon, nitrogen and water use efficiency, ecological service value of winter wheat. Henan Normal University.
16. Ma C, 2018. Effects of combined application of inorganic fertilizer and organic manure on nitrogen fertilization rate wheat grain yield and soil environment on the loess plateau dryland. Northwest A & F University.
17. Meng F H, Gao J L, Yu X F, Wang Z G, Hu S P, Qing G E, Sun J Y, Qu J W, 2018. Inprovent of biochemical property of surface soil by combined application of biochar with nitrogen fertilizer. Journal of Plant Nutrition and Fertilizers, 4(5): 1214-1226.
18. Song D L, Xi X Y, Huang S M, Zhang S Q, Yuan X M, Huang F S, Liu Y, Wang X B, 2017. Effects of combined application of straw biochar and nitrogen on soil carbon and nitrogen contents and crop yields in a fluvo-aquic soil. Journal of Plant Nutrition and Fertilizer, 23(2): 369–379.
19. Tao Y Y, Jin M J, Tang Y L, Zhu X L, Lu C Y, Wang H H, Shi L L, Zhou X W, Shen M X, 2017. Partial nitrogen fertilizer substitution by aquatic plant compost to improve rice yield and paddy soil fertility. Transactions of the Chinese Society of Agricultural Engineering. 33(18): 196-202.
20. Tian X F, 2017. Effects of controlled release nitrogen, potassium fertilizers and biochar on cotton growth and soil nutrient status, Shandong Agricultural University.
21. Wang Y C, Cao S H, Chu L, Lu Y, Zhang F F, Hou J X, 2010. Effects of different proportion of biogas slurry and nitrogen fertilizer application on dry matter accumulation of silage maize and soil fertility. Acta Agriculturae Boreali-occidentalis Sinica, 19(9): 163-167.
22. Wang Q J, 2012. Effects of application of mixed chemical fertilizer with composts on crop growth and soil fertility in rice-wheat rotation system. Nanjing Agricultural University.
23. Wang N, 2015. Effect of N dosage on the soil microbial characteristics and its organic C components from a corn field. Jilin Agricultural University.
24. Wang R, Hu Y X, Wang, Y, Ali S, Liu Q F, Guo S L, 2019. Nitrogen application increases soil respiration but decreases temperature sensitivity: combined effects of crop and soil properties in a semiarid agroecosystem. Geoderma, 353: 320-330.
25. Wang Y L, Wu P N, Li P F, Wang X N, Zhu X, 2019. Effects of organic manure combined with nitrogen fertilizer on spring maize yield and soil fertility under drip irrigation. Acta Agronomic Sinica, 45(8): 1230-1237.
26. Wang Z, Wang Z, Luo Y, Zhan Y N, Meng Y L, Zhou Z G, 2020. Biochar increases ^15^N fertilizer retention and indigenous soil N uptake in a cotton-barley rotation system. Geoderma, 357: 113944.
27. Xie Y Q, 2015. Effect of nitrogen management modes on soil environment in double rice cropping system. Sichuan Agricultural University.
28. Yang Z Z, 2019. Effects of organic fertilizer substitution on physical and chemical properties of black soil and maize yield, Northeast Agricultural University.
29. Yu X Y, Zhai B N, Jin Z Y, Li Y G, Wang Y, Zhang H Q, Wang Z H, 2015. Effect of combined application of organic and inorganic fertilizers on winter wheat yield, water, and fertilizer use efficiency and soil fertility in dryland. Journal of sol and water conservation, 29(5):320-32
30. Yu H L, Ling N, Wang T T, Zhu C, Wang Y, Wang S J, Gao Q, 2019. Responses of soil biological traits and bacterial communities to nitrogen fertilization mediate maize yields across three soil types. Soil & Tillage Research, 185: 61-69.
31. Yuan J J, Tong Y A, Lu S H, Yuan G J, 2017. Effects of biochar and nitrogen fertilizer application on soil fertility and jujube yield and quality. Journal of Plant Nutrition and Fertilizers, 23(2): 468-475.
32. Yuan T Y, 2017. Study on Yield Increasing Effect and the mechanism of combined application of humic acid and reducing fertilizer nitrogen on winter wheat summer maize rotation system.
33. Zhang D, 2014. Research on the effects of inorganic-organic fertilizer incorporation under rice-wheat rotation system in Hubei province. Nanjing Agricultural University.
34. Zhang Y Q, 2015. Effects of long term combination application of different organic materials and nitrogen fertilizer on carbon nitrogen and microbial characteristics of soil. Henan Agricultural University.
35. Zhang X L, 2017. Effect of different reducing nitrogen fertilization on soil environment in double rice cropping system. Sichuan Agricultural University.
36. Zuo W G, 2016. Effects of nitrogen management on grain yield and nitrogen use of double cropping rice system with all rice straw returned to the field. Yangzhou University.

**Table.S2. Latitude (lat) and longitude (lon) of the 46 study sites used in this study.**

| Number | Lon | Lat |
| --- | --- | --- |
| 1 | 113.38 | 28.53 |
| 2 | 115.13 | 28.25 |
| 3 | 115.13 | 28.25 |
| 4 | 113.72 | 35.28 |
| 5 | 104.75 | 35.47 |
| 6 | 116.81 | 35.59 |
| 7 | 108.08 | 34.29 |
| 8 | 116.18 | 28.36 |
| 9 | 113.73 | 30.05 |
| 10 | 109.60 | 35.18 |
| 11 | 108.08 | 34.27 |
| 12 | 114.51 | 35.64 |
| 13 | 120.35 | 37.58 |
| 14 | 107.67 | 35.22 |
| 15 | 118.83 | 32.03 |
| 16 | 114.90 | 36.70 |
| 17 | 124.00 | 43.33 |
| 18 | 124.28 | 39.42 |
| 19 | 85.68 | 44.38 |
| 20 | 116.75 | 33.62 |
| 21 | 111.88 | 26.75 |
| 22 | 115.87 | 28.68 |
| 23 | 102.88 | 37.91 |
| 24 | 115.13 | 28.26 |
| 25 | 126.49 | 43.96 |
| 26 | 112.45 | 32.77 |
| 27 | 109.60 | 35.18 |
| 28 | 119.23 | 31.65 |
| 29 | 113.67 | 34.78 |
| 30 | 120.91 | 31.60 |
| 31 | 121.91 | 32.60 |
| 32 | 112.08 | 30.87 |
| 33 | 116.66 | 35.02 |
| 34 | 117.99 | 36.98 |
| 35 | 115.74 | 37.73 |
| 36 | 114.05 | 32.99 |
| 37 | 111.77 | 30.49 |
| 38 | 121.50 | 43.86 |
| 39 | 110.56 | 40.60 |
| 40 | 114.92 | 35.75 |
| 41 | 117.27 | 31.87 |
| 42 | 120.60 | 31.42 |
| 43 | 106.87 | 37.63 |
| 44 | 107.41 | 34.59 |
| 45 | 124.48 | 43.25 |
| 46 | 124.08 | 43.35 |

**Table. S3 mean annual temperature (MAT), mean annual precipitation (MAP), accumulated temperature above 10℃ (MATA) of 46 study sites used in this study.**

| **MAT(℃)** | **MAP (mm)** | **MATA (℃)** |
| --- | --- | --- |
| 16.5 | 1389 | 6539 |
| 16.6 | 1551 | 6001 |
| 18.2 | 1700 | 5860 |
| 15.7 | 1100 | 5750 |
| 17.2 | 1680 | 5644 |
| 17.14 | 1668 | 5432.8 |
| 17.1 | 1607 | 5407 |
| 17.5 | 1600 | 5400 |
| 16 | 1200 | 5350 |
| 16．5 | 1041.8 | 5300 |
| 15 | 805.8 | 5296.7 |
| 15.6 | 1085 | 5190 |
| 15.7 | 940 | 5100 |
| 15.4 | 1054 | 4933.7 |
| 14.4 | 640.9 | 4925 |
| 15.2 | 1058.8 | 4859.6 |
| 16.4 | 1363.5 | 4820 |
| 15 | 960 | 4776 |
| 16.6 | 1315 | 4753.6 |
| 14.6 | 557.2 | 4700 |
| 13.8 | 673 | 4605.5 |
| 13.6 | 750 | 4571.9 |
| 15 | 700 | 4500 |
| 12.5 | 582.8 | 4500 |
| 13.2 | 494 | 4472.0 |
| 11.5 | 577 | 4448 |
| 11.4 | 577 | 4312 |
| 13.37 | 640 | 4485.1 |
| 15 | 626.11 | 3829 |
| 12.18 | 559 | 3650 |
| 12.9 | 550 | 3650 |
| 15.1 | 497.5 | 3553 |
| 13.4 | 600 | 3500 |
| 8 | 213 | 3463.5 |
| 17 | 1570 | 3400 |
| 6.6 | 350 | 3200 |
| 7.8 | 158 | 3200 |
| 5.8 | 474 | 3046.8 |
| 9.4 | 560 | 2994 |
| 8.5 | 262.9 | 2965 |
| 22.4 | 280 | 2949.9 |
| 22.14 | 290 | 2949.19 |
| 3.9 | 690.4 | 2700 |
| —0.1 | 570 | 2571.2 |





Fig.S1 Relationship between mean annual temperature (MAT), mean annual precipitation (MAP), accumulated temperature above 10℃ (MATA) and the natural logarithm of the response ratio (lnRR) of soil organic carbon (SOC), total nitrogen (TN), and the ratio of SOC to TN (SOC:TN. The black solid line shows the relationship between the lnRR of SOC, TN, and SOC:TN and environmental variables of the all database.

Table S4. Checklist of the present study

| **Section and Topic** | **Item #** | **Checklist item** | **Location where item is reported** |
| --- | --- | --- | --- |
| **TITLE** | | |  |
| Title | 1 | Identify the report as a systematic review. | 1 |
| **ABSTRACT** | | |  |
| Abstract | 2 | See the PRISMA 2020 for Abstracts checklist. | 1 |
| **INTRODUCTION** | | |  |
| Rationale | 3 | Describe the rationale for the review in the context of existing knowledge. | 2,3 |
| Objectives | 4 | Provide an explicit statement of the objective(s) or question(s) the review addresses. | 4 |
| **METHODS** | | |  |
| Eligibility criteria | 5 | Specify the inclusion and exclusion criteria for the review and how studies were grouped for the syntheses. | 18 |
| Information sources | 6 | Specify all databases, registers, websites, organisations, reference lists and other sources searched or consulted to identify studies. Specify the date when each source was last searched or consulted. | 17 |
| Search strategy | 7 | Present the full search strategies for all databases, registers and websites, including any filters and limits used. | 18 |
| Selection process | 8 | Specify the methods used to decide whether a study met the inclusion criteria of the review, including how many reviewers screened each record and each report retrieved, whether they worked independently, and if applicable, details of automation tools used in the process. | 18 |
| Data collection process | 9 | Specify the methods used to collect data from reports, including how many reviewers collected data from each report, whether they worked independently, any processes for obtaining or confirming data from study investigators, and if applicable, details of automation tools used in the process. | 18 |
| Data items | 10a | List and define all outcomes for which data were sought. Specify whether all results that were compatible with each outcome domain in each study were sought (e.g. for all measures, time points, analyses), and if not, the methods used to decide which results to collect. | 19 |
|  | 10b | List and define all other variables for which data were sought (e.g. participant and intervention characteristics, funding sources). Describe any assumptions made about any missing or unclear information. | 19 |
| Study risk of bias assessment | 11 | Specify the methods used to assess risk of bias in the included studies, including details of the tool(s) used, how many reviewers assessed each study and whether they worked independently, and if applicable, details of automation tools used in the process. | 20 |
| Effect measures | 12 | Specify for each outcome the effect measure(s) (e.g. risk ratio, mean difference) used in the synthesis or presentation of results. | 20 |
| Synthesis methods | 13a | Describe the processes used to decide which studies were eligible for each synthesis (e.g. tabulating the study intervention characteristics and comparing against the planned groups for each synthesis (item #5)). | 18 |
|  | 13b | Describe any methods required to prepare the data for presentation or synthesis, such as handling of missing summary statistics, or data conversions. | 20 |
|  | 13c | Describe any methods used to tabulate or visually display results of individual studies and syntheses. | 20 |
|  | 13d | Describe any methods used to synthesize results and provide a rationale for the choice(s). If meta-analysis was performed, describe the model(s), method(s) to identify the presence and extent of statistical heterogeneity, and software package(s) used. | 20 |
|  | 13e | Describe any methods used to explore possible causes of heterogeneity among study results (e.g. subgroup analysis, meta-regression). | 20 |
|  | 13f | Describe any sensitivity analyses conducted to assess robustness of the synthesized results. | 20 |
| Reporting bias assessment | 14 | Describe any methods used to assess risk of bias due to missing results in a synthesis (arising from reporting biases). | na |
| Certainty assessment | 15 | Describe any methods used to assess certainty (or confidence) in the body of evidence for an outcome. | 20 |
| **RESULTS** | | |  |
| Study selection | 16a | Describe the results of the search and selection process, from the number of records identified in the search to the number of studies included in the review, ideally using a flow diagram. | 18 |
|  | 16b | Cite studies that might appear to meet the inclusion criteria, but which were excluded, and explain why they were excluded. | 18 |
| Study characteristics | 17 | Cite each included study and present its characteristics. | 18 |
| Risk of bias in studies | 18 | Present assessments of risk of bias for each included study. | 19 |
| Results of individual studies | 19 | For all outcomes, present, for each study: (a) summary statistics for each group (where appropriate) and (b) an effect estimate and its precision (e.g. confidence/credible interval), ideally using structured tables or plots. | 4-16 |
| Results of syntheses | 20a | For each synthesis, briefly summarise the characteristics and risk of bias among contributing studies. | 4 |
|  | 20b | Present results of all statistical syntheses conducted. If meta-analysis was done, present for each the summary estimate and its precision (e.g. confidence/credible interval) and measures of statistical heterogeneity. If comparing groups, describe the direction of the effect. | Fig.2,Fig.3 |
| Reporting biases | 21 | Present assessments of risk of bias due to missing results (arising from reporting biases) for each synthesis assessed. | na |
| Certainty of evidence | 22 | Present assessments of certainty (or confidence) in the body of evidence for each outcome assessed. | 4-16 |
| **DISCUSSION** | | |  |
| Discussion | 23a | Provide a general interpretation of the results in the context of other evidence. | 16 |
|  | 23b | Discuss any limitations of the evidence included in the review. | na |
| **OTHER INFORMATION** | | |  |
| Registration and protocol | 24 | Provide registration information for the review, including register name and registration number, or state that the review was not registered. | na |
| Support | 25 | Describe sources of financial or non-financial support for the review, and the role of the funders or sponsors in the review. | 21 |
| Competing interests | 26 | Declare any competing interests of review authors. | 21 |
| Availability of data, code and other materials | 27 | Report which of the following are publicly available and where they can be found: template data collection forms; data extracted from included studies; data used for all analyses; analytic code; any other materials used in the review. | 17 |
